# Trends in Cause-Specific Injury Mortality in China in 2005-2019: Longitudinal Observational Study

**DOI:** 10.2196/47902

**Published:** 2023-09-15

**Authors:** Zixiang Ji, Hengjing Wu, Rongyu Zhu, Lu Wang, Yuzhu Wang, Lijuan Zhang

**Affiliations:** 1 Clinical Center for Intelligent Rehabilitation Research, Shanghai YangZhi Rehabilitation Hospital Tongji University School of Medicine, Tongji University Shanghai China

**Keywords:** reverse, age-standardized mortality rate, injury, suicide, trend, potential years of life lost, average years of life lost, crude mortality rate, falls, older adults, young adults

## Abstract

**Background:**

Over the last few decades, although the age-standardized mortality rate (ASMR) of injury has shown a significant declining trend in China, this pattern has dramatically reversed recently.

**Objective:**

We aimed to elucidate the geographical, demographic, and temporal trends of cause-specific injuries, the reversal phenomenon of these trends, and the fluctuations of injury burden from 2005 to 2019 in China.

**Methods:**

A longitudinal observational study was performed using the raw data of injury deaths in the National Cause-of-Death surveillance data provided by the disease surveillance points system in 2005-2019. The cause-specific injuries were divided into disparate subgroups by sex, age, urban/rural region, and eastern/central/western areas of China. The burden of injury was assessed using potential years of life lost (PYLL), average years of life lost (AYLL), and PYLL rate (PYLLR). Temporal trends of mortality rates and burden were evaluated using best-fitting joinpoint models.

**Results:**

Injury deaths accounted for 7.51% (1,156,504/15,403,835) of all-cause deaths in China in 2005-2019. The crude mortality rate of all-cause injury was 47.74 per 100,000 persons. The top 3 injury types (traffic accident, falls, and suicide) accounted for 70.57% (816,145/1,156,504) of all injury-related deaths. The ASMR of all-cause injury decreased (*P*=.003), while the crude mortality rate remained unchanged (*P*=.52) during 2005-2019. A significant reverse trend in ASMR of all-cause injury was observed in urban older adults since 2013, mainly due to the inverted trend in injuries from falls. A reverse trend in ASMR of suicide was observed among individuals aged 10-24 years, with notable increases by 35.18% (annual percentage change 15.4%, 95% CI 4.1%-28.0%) in men since 2017. The AYLL and PYLLR of all-cause injury among older adults showed consistent ascending trends from 2005 to 2019 (average annual percentage change [AAPC] 6.1%, 95% CI 5.4%-6.9%, 129.04% increase for AYLL; AAPC 5.4%, 95% CI 2.4%-8.4%, 105.52% increase for PYLLR). The AYLL due to suicide for individuals aged 10-24 years showed a considerable upswing tendency (AAPC 0.5%, 95% CI 0.4%-0.7%, 8.02% increase).

**Conclusions:**

Although the ASMR of all-cause injury decreased in China from 2005 to 2019, the trend in suicide among adolescents and young adults and falls among older adults has been on the rise in recent years. Interventions should be encouraged to mitigate the cause-specific burdens of injury death.

## Introduction

Injury continues to pose a significant public health risk factor worldwide with a high mortality rate, and it contributes to more than 5 million deaths annually [1], accounting for about 9% of global mortality [2]. In China, injury is the fifth leading cause of death [3], accounting for almost 500,000 deaths and 300 million injuries per year [4], and the massive cost of health care amounts to US $9.6 billion annually [5]. Injury-related long-term disability sequelae [6] and deaths could have important negative effects on families, societies, and governments, including decline of the economy, destruction of social production, and restriction of the growth of the country’s overall strengths to some extent.

With its reform and opening-up policy as well as its membership in the World Health Organization, China has experienced an unprecedented rise in motorized vehicles and a major transformation in the mode of travel and transportation [7]. The incidence of injuries from motor vehicle crashes has substantially increased, particularly in the rural regions of China, where the conflict between the proliferation of cars and the inability to keep up with road traffic management measures has greatly contributed to the frequency of motor vehicle crashes [5]. Meanwhile, drowning has become the leading cause of childhood injury mortality [5] due to the high percentage of children left without supervision and care because of increased working opportunities and income for parents in urban regions. The advancement of ideologies has lagged economic growth, and suicide among rural women has become the leading cause of injury death [8]. However, with the introduction of the “Health China 2030 plan,” the concept of health has gradually been reinforced, and cause-specific injury has shown a meaningful declining trajectory [9] with the strengthening of health education, improvement in the health service system, and optimization of related policies and measures. Despite advancements in health policy initiatives in China, differences in injury mortalities in the underprivileged communities require further investigations. The origins of injury-related deaths in rural regions, particularly those caused by suicide, may alter significantly as time goes on, globalization increases, and ideologies shift drastically.

This study investigates the association of the geographical, demographic, and temporal trends of cause-specific injuries with the magnitude and patterns of injury mortality in mainland China during 2005-2019. Temporal and age-specific trends were analyzed to explore the intricacies of injury mortality in various periods, geographies, and populations. Our findings can serve as a foundation for further research and provide the government and administration with scientific evidence for the formulation of relevant government policies and execution of judgments to lower the risks of injury and death.

## Methods

### Source of Data

Injury death data were derived from the National Cause-of-Death Surveillance data set, which was recorded and rechecked for a fixed period by the disease surveillance points (DSP) system established in 1978. By 2013, the number of points had increased to 605 with a population of more than 300 million Chinese residents, accounting for more than 24% of the whole national population [7]. The strict procedures [10] involved in collecting and evaluating data were implemented between upper and lower administrative regions to ensure the data’s completeness, authenticity, and reliability. Information on individual deaths in all population catchment areas has been reported in real time by using an internet-based reporting system by the local Centers for Disease Control and Prevention (CDC) since 2008. Local CDCs also check the coding and internal logic of items reported on death certificates, and then death data are subsequently reported to the national CDC. Following a review and correction during the previous quarter, the Chronic Disease Center of the CDC finally receives all death numbers from the monitoring sites. Any unreliable reports are addressed by thoroughly analyzing medical records or by conducting verbal autopsies. Additionally, every 3 years, a regular survey of a nationally representative sample is conducted at each disease monitoring point location to collect data that correct underreporting. Together, the DSPs provided a nationally representative picture of mortality in China. The International Classification of Diseases tenth revision was used to code injuries based on their underlying causes of mortality. In general, the injuries (V0-Y89) are split into 2 categories: intentional injuries and unintentional injuries. The formed category consists of suicide and other intentional injuries, while the latter consists of traffic accidents, poisoning, falls, fires, drowning, and other unintentional injuries. We disaggregated the data by subgroups such as sex (men and women), region (urban and rural), area (eastern, central, and western), and age (≤39 years, 40-64 years, and ≥65 years).

### Data Analysis

The crude mortality rate (CMR), age-standardized mortality rate (ASMR) and 95% CIs, and rates of rank and proportion of death causes were calculated every year from 2005 to 2019. For overall and cause-specific injury, risk ratio (RR) values for CMR and ASMR were calculated separately for different sexes, regions, and areas. The age-period-cohort model was implemented to develop independent effect estimates of age, period, and birth cohort on injury and suicide mortality to analyze period- and cohort-related risks and longitudinal and cross-sectional age trends. Populations were divided into 5-year intervals from 2005 to 2019. The longitudinal age curve comprised consecutive 5-year age segments from 0-4 years to 85+ years for all-cause injury and from 5-9 years to 85+ years for suicide due to virtually no suicide deaths among those younger than 5 years. The RR values and the estimable parameters and functions concerning the age-period-cohort model should be reanalyzed by Fisher exact test if the Wald chi-square test did not meet the criteria. Temporal and age-specific trends both reflected changes in injury mortality. Joinpoint regression analysis was implemented to log-transform the CMR and ASMR, and the optimum yearly trend curve and rate of variation were then exhibited using the Grid Search method, Monte Carlo permutation tests, and Modified Bayesian Information Criterion. Annual percentage change (APC) showed the rate for certain segments within the study period, while average APC (AAPC) represented the average change rate from 2005 to 2019, and 95% CI suggested statistical significance. The potential years of life lost (PYLL), average years of life lost (AYLL), and PYLL rate (PYLLR) (Multimedia Appendix 1) were indicators of the injury burden that quantified the impact of premature mortality on PYLL. Epidata 3.1 (The Epidata Association, Odense) and Excel (version 2019; Microsoft) were utilized for data entry and management. Joinpoint (Joinpoint Regression Program version 4.9.1.0; National Cancer Institute) was applied to present the trends of injury mortality changes under best-fitting joinpoint models. The age-period-cohort online tool [11] (National Cancer Institute) provided the calculated parameters of the age-period-cohort model. R software (version 4.2.2; MathSoft) was used for statistical analysis. The results were considered significant when *P* values were less than .05 in 2-sided tests.

### Ethics Approval

This research received ethics approval from the ethics committee of the School of Medicine, Tongji University, Shanghai (approval 2023tjdxsy037).

## Results

### Basic Information on Injury Deaths in China During 2005-2019

A total of 2,414,759,166 person-years recorded by DSPs were included in this investigation from 2005 to 2019. The number of injury deaths was 1,156,504 (men:women=2.1:1.0), accounting for 7.51% of all-cause deaths (n=15,403,835) within the study period. The details of the annual demographic data are presented in Table S2 of Multimedia Appendix 2, and the entire aggregate of information concerning injuries in these 15 years is shown in Tables S3 and S4 of Multimedia Appendix 2. The CMR of all injuries was 47.74 per 100,000 (95% CI 47.65-47.82 per 100,000). Traffic accidents (16.57 per 100,000) was the leading cause of injury-related deaths, followed by falls (9.66 per 100,000), suicide (7.46 per 100,000), drowning (3.75 per 100,000), poisoning (3.00 per 100,000), and fires (0.64 per 100,000). The 3 major causes of death accounted for 70.57% (1,156,504/15,403,835) of all injury-related deaths, with ASMR following the same pattern. In the whole population, the CMRs and ASMRs of all-cause injury in men were significantly higher than those in women (RR_men vs women_ 2.024, 95% CI 2.016-2.032 for CMR; RR_men vs women_ 2.397, 95% CI 2.389-2.407 for ASMR). The mortality rates of all-cause injury in rural residents were generally higher than those in urban residents (RR_rural vs urban_ 1.492, 95% CI 1.486-1.499 for CMR; RR_rural vs urban_ 1.577, 95% CI 1.570-1.584 for ASMR). Western area residents had considerably higher mortality rates of all-cause injury than eastern area residents (RR_western vs eastern_ 1.179, 95% CI 1.174-1.185 for CMR; RR_western vs eastern_ 1.408, 95% CI 1.401-1.415 for ASMR) and central area residents (RR_western vs central_ 1.146, 95% CI 1.140-1.151 for CMR; RR_western vs central_ 1.214, 95% CI 1.208-1.219 for ASMR). We also ranked each injury type according to disparate geographical and economic regions (Figures S1 and S2 of Multimedia Appendix 2). Traffic accidents and suicide were the top 2 injury deaths in the central areas of China, while traffic accidents and falls were the leading causes of injury in other regions.

### Temporal Trends of Injuries in China From 2005 to 2019

**Figure 1:**
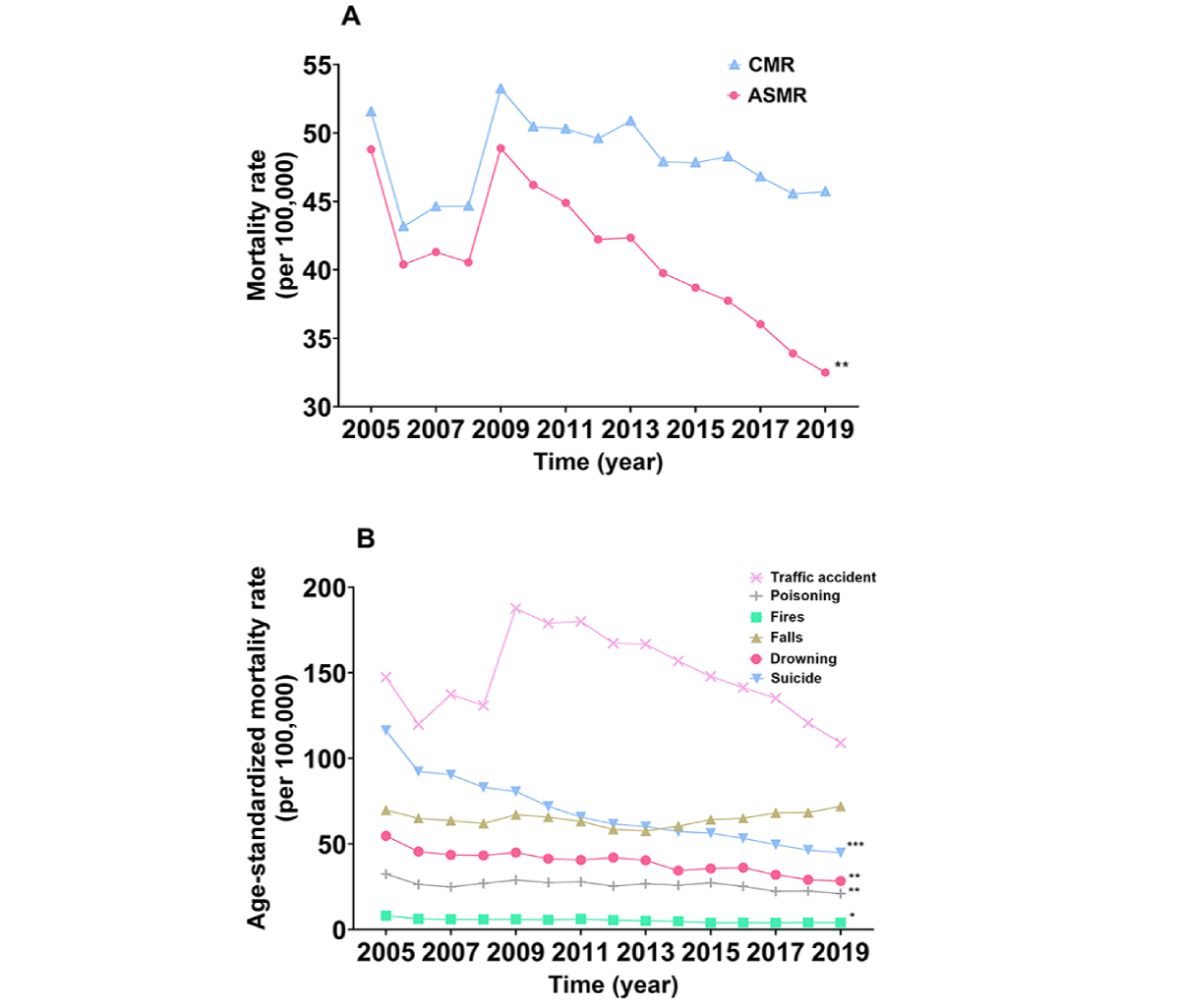
Trends in the mortality rates of all-cause injury and disparate types of injury in China in 2005-2019. A. Trends in crude mortality rates and age-standardized mortality rates per 100,000 cases of all-cause injury in 2005-2019. B. Trends in age-standardized mortality rates per 100,000 cases of disparate types of injury in 2005-2019. ASMR: age-standardized mortality rate; CMR: crude mortality rate. *P<.05, **P<.01, ***P<.001.

**Figure 2:**
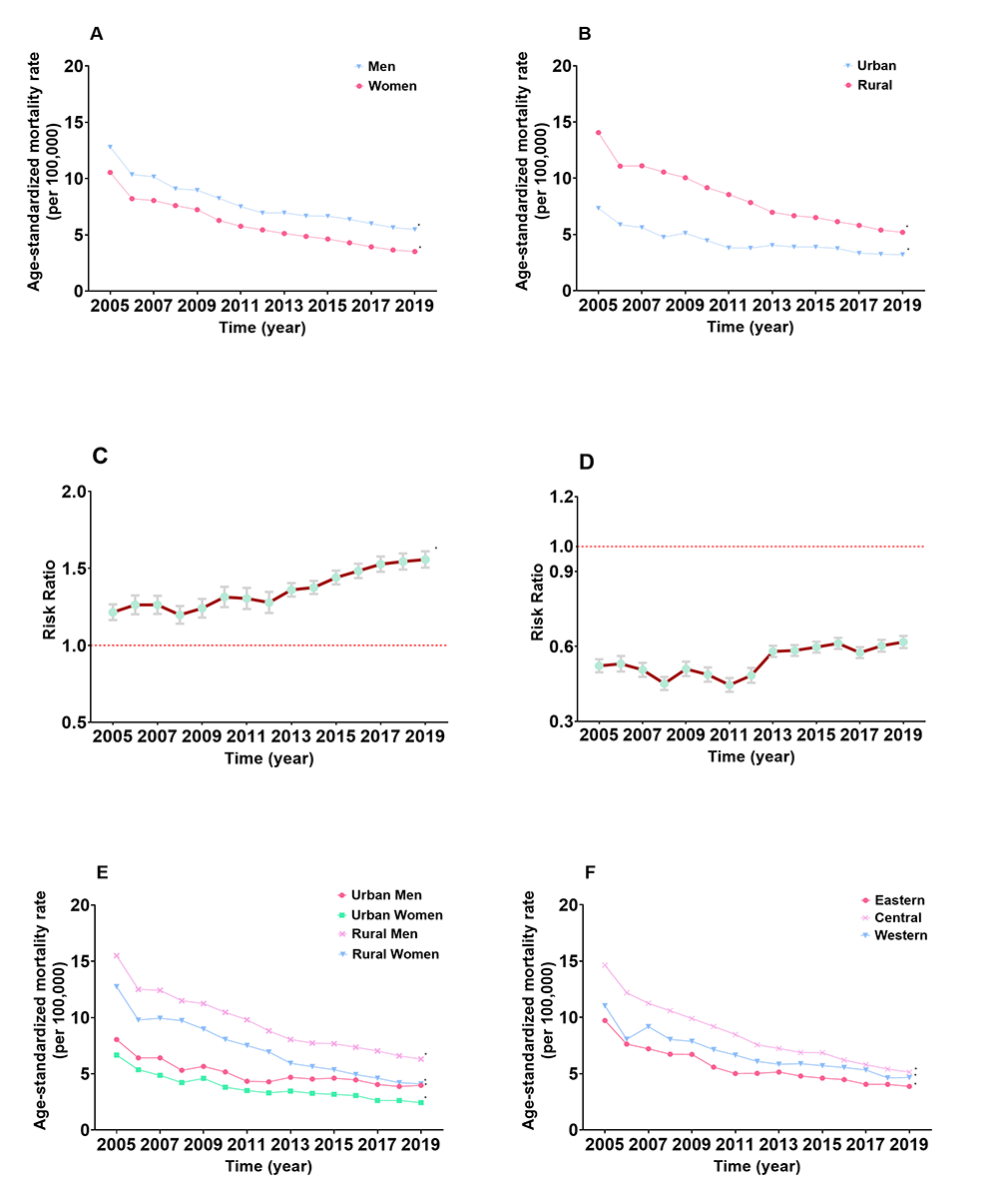
Trends in age-standardized mortality rates per 100,000 cases and risk ratio of suicide in the whole population in China in 2005-2019. A. The trend in age-standardized mortality rate by sex. B. The trend in age-standardized mortality rate by region. C. The trend in age-standardized mortality rate risk ratio of men to women. D. The trend in age-standardized mortality rate risk ratio of urban regions to rural regions. E. The trend in age-standardized mortality rate by sex and region. F. The trend in age-standardized mortality rate by area. *P<.001.

The ASMR of all-cause injury decreased by 33.42% (AAPC –2.6%, 95% CI –4.3% to –0.9%), while the CMR remained stable (AAPC –0.7%, 95% CI –2.8% to 1.4%) from 2005 to 2019 (Figure 1). The ASMRs of suicide decreased by 61.40% (AAPC –6.3%, 95% CI –7.8% to –4.9%), drowning decreased by 48.26% (AAPC –4.6%, 95% CI –7.4% to –1.7%), fires decreased by 50.73% (AAPC –4.0%, 95% CI –7.5% to –0.3%), and poisoning decreased by 35.12% (AAPC –2.9%, 95% CI –4.9% to –0.8%), while traffic accidents and falls exhibited no significant change during this period (both *P*>.05). Trends of injury ASMR by sex, region, area, age, and type are shown in Table S7 of Multimedia Appendix 2. Briefly, the ASMR of all-cause injury decreased by 31.85% (AAPC –2.4%, 95% CI –4.2% to –0.5%) in men and by 36.67% in women (AAPC –3.1%, 95% CI –4.4% to –1.7%). The ASMR of all-cause injury in rural residents decreased by 33.85% (AAPC –2.8%, 95% CI –5.4% to –0.1%), while the ASMR of urban residents remained unchanged (AAPC –2.3%, 95% CI –6.7% to 2.2%). The ASMR of all injuries decreased by 31.79% in the eastern areas (AAPC –2.4%, 95% CI –4.9% to 0.1%), 38.90% in the central areas (AAPC –3.3%, 95% CI –4.8% to –1.6%), and 28.68% in the western areas (AAPC –2.0%, 95% CI –3.9% to 0.0%). Despite a substantial downtrend in overall suicides between 2005 and 2019, various subgroups showed distinct patterns (Figure 2, Table S8 of Multimedia Appendix 2). The overall ASMR of suicide decreased in men by 57.19% (AAPC –5.5%, 95% CI –7.3% to –3.7%) and by 66.62% in women (AAPC –7.4%, 95% CI –8.6% to –6.1%). The ASMR of suicide in men was significantly higher than that in women (RR 1.380, 95% CI 1.367-1.394) and the trend of RR increased by 28.25% (AAPC 1.8%, 95% CI 1.1%-2.5%) during the whole study period, particularly after 2009 (APC 2.4%, 95% CI 1.8%-3.1%). Although the ASMR of suicide decreased in the rural regions by 63.09% (AAPC –6.6%, 95% CI –7.4% to –5.8%) and by 56.35% in the urban regions (AAPC –5.5%, 95% CI –8.3% to –2.7%), the ASMR of suicide in rural residents was higher than that in urban residents (RR 1.767, 95% CI 1.747-1.788). The ratio of urban region to rural region suicide ASMRs showed a generally increasing tendency, inching closer to 1, especially after 2008 (36.93% increase, APC 2.8%, 95% CI 1.3%-4.3%). Further analysis revealed that the highest ASMR of suicide was continuously reported in rural men, while the ASMR of suicide in urban women was consistently minimum. Contrary to all-cause injury, suicide presented the highest ASMR in the central areas of China, where there was also the steepest decline in the slope in 2005-2019 (64.94% decrease in the central areas, AAPC –7.0%, 95% CI –8.4% to –5.5%), followed by the eastern areas (60.25% decrease, AAPC –6.2%, 95% CI –8.4% to –3.9%) and the western areas (57.79% decrease, AAPC –5.7%, 95% CI –8.4% to –2.8%).

### Temporal Reverse Trends of Injuries in China From 2005 to 2019

**Figure 3:**
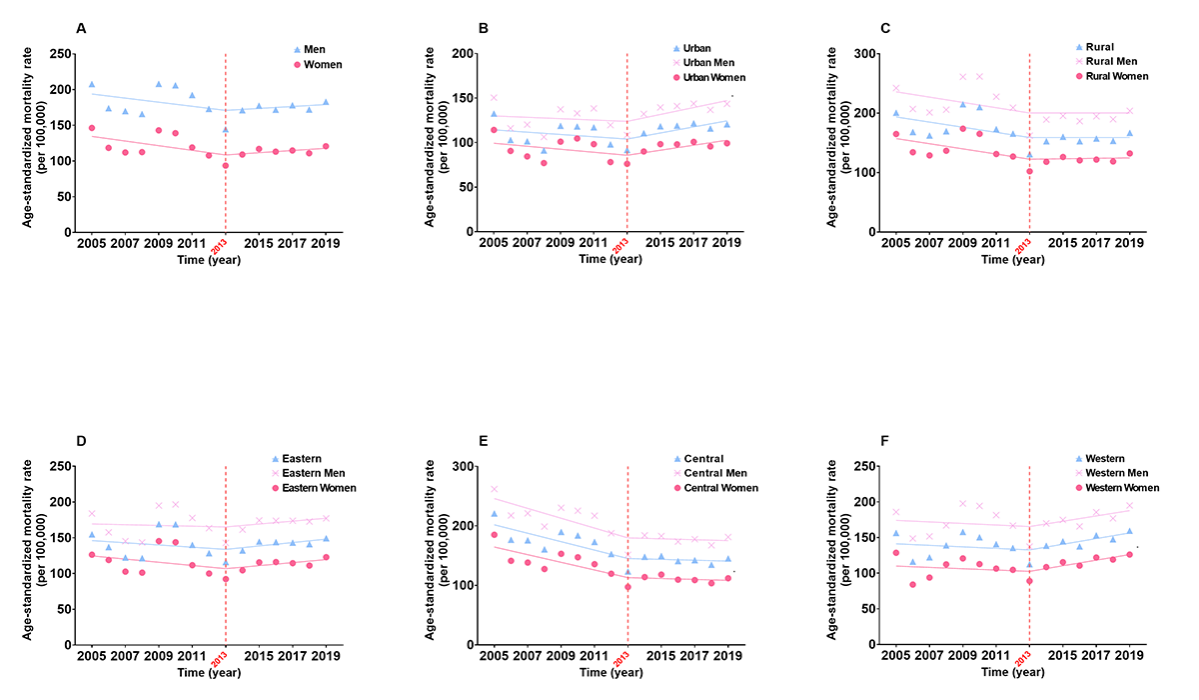
Trends in age-standardized mortality rates per 100,000 older adults in China in 2005-2019. A. The trend in age-standardized mortality rate among older adults by sex. B. The trend in age-standardized mortality rate of older adults by urban region and sex. C. The trend in age-standardized mortality rate of older adults by rural region and sex. D. The trend in age-standardized mortality rate of older adults by eastern area and sex. E. The trend in age-standardized mortality rate of older adults by central area and sex. F. The trend in age-standardized mortality rate of older adults by western area and sex. *P<.01 (for trends in 2013-2019).

**Figure 4:**
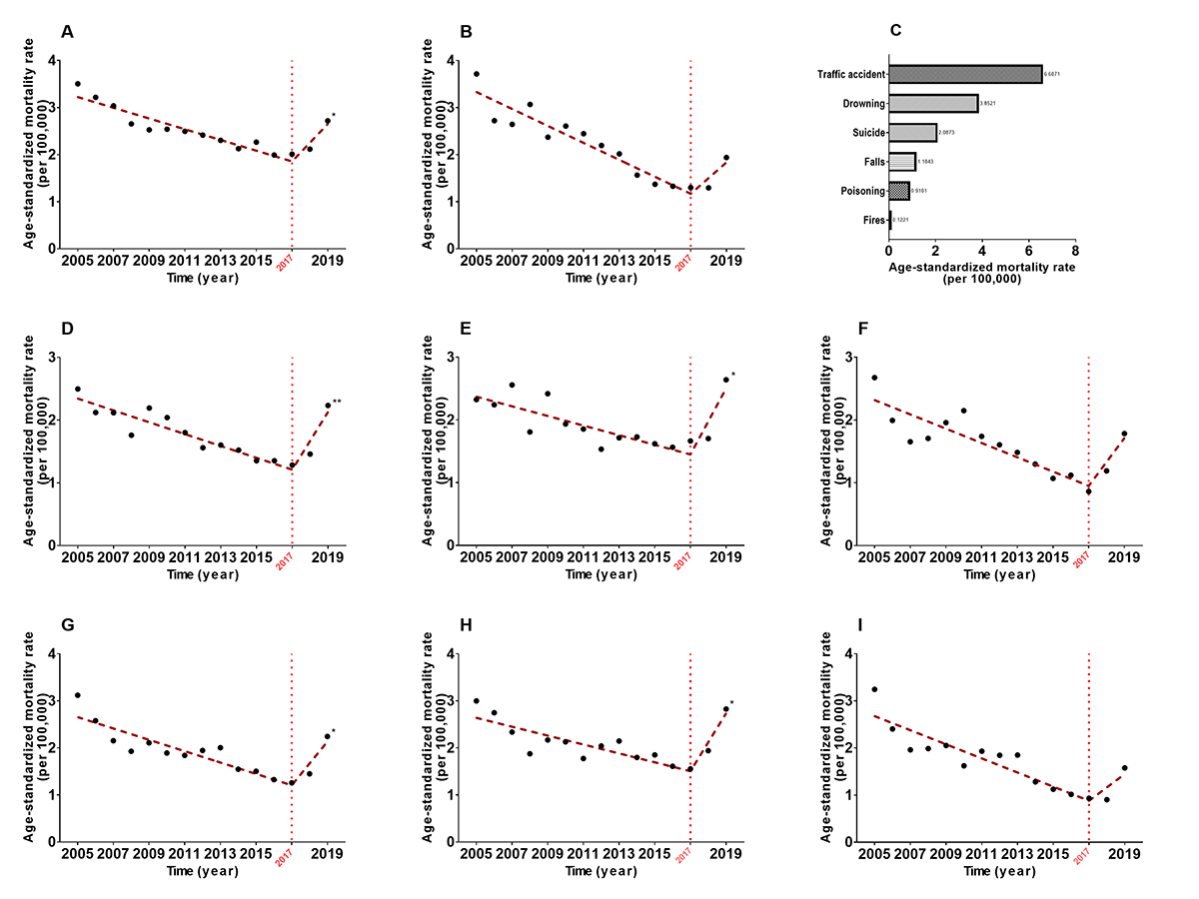
Trends of suicide among young individuals aged 10-24 years in the whole population as well as urban and eastern area populations of China and the ranks of the leading causes of injury among young individuals aged 10-24 years in 2005-2019. A. The trend of suicide among males aged 10-24 years. B. The trend of suicide among females aged 10-24 years. C. The ranks of the leading causes of injury among young individuals aged 10-24 years. D. The trend of suicide among young individuals aged 10-24 years in urban regions. E. The trend of suicide among males aged 10-24 years in urban regions. F. The trend of suicide among females aged 10-24 years in urban regions. G. The trend of suicide among young individuals aged 10-24 years in eastern areas. H. The trend of suicide among males aged 10-24 years in eastern areas. I. The trend of suicide among females aged 10-24 years in eastern areas. *P<.05, **P<.01 (for trends in 2017-2019).

**Figure 5:**
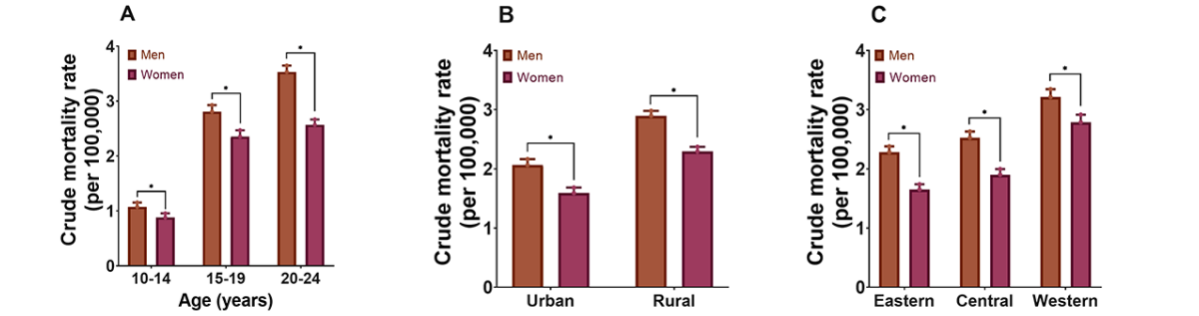
The crude mortality rate per 100,000 suicide cases of both sexes within the disparate subgroups among adolescents and young adults aged 10-24 years in China in 2005-2019. A. Crude mortality rates in the different age groups in China in 2005-2019. B. Crude mortality rates in urban and rural China in 2005-2019. C. Crude mortality rates in eastern, central, and western areas of China in 2005-2019. *P<.001.

Several age groups showed reversals in the temporal trends for all-cause injury and suicide ASMRs in contrast to the overall population’s patterns. We divided the participants into 3 age groups: ≥65 years old (older adults), 40-64 years old, and ≤39 years old. The ASMRs of all-cause injury in the ≤39 years and 40-64 years populations decreased by 46.69% and 31.40%, respectively, but the decrease was less obvious in the older adult populations (*P=*.93). Among the older adult populations, a reverse trend of all-cause injury was observed since 2013. Therefore, we further analyzed the underlying situations and present the results in Figure 3. These reverse trends occurred since 2013, especially among men in the urban regions (32.38% increase, APC 1.7%, 95% CI 0.6%-2.8%) and women in the western areas of China (41.73% increase, APC 1.9%, 95% CI 0.8%-2.9%), although there were increasing tendencies across all areas, as well as all men and women except for the central area residents. Among older adults, falls were the leading cause of the reversal trends. The ASMRs of falls showed significantly increasing tendencies since 2013 among all subgroups, even among the central area residents (All *P*<.05, Figure S3 of Multimedia Appendix 2). After stratification by age, we found the trend in ASMR of suicide among adolescents and young adults aged 10-24 years had reversed recently (Figure 4, Figure S4 of Multimedia Appendix 2). Detailed analysis revealed a noticeable increase by 35.18% (APC 15.4%, 95% CI 4.1%-28.0%) in males aged 10-24 years since 2017 (Figure 4). Remarkable increases in the urban (73.98% increase, APC 32.4%, 95% CI 9.9%-59.3%) and eastern area (78.32% increase, APC 34.7%, 95% CI 6.8%-69.9%) residents were found in this study, which might be contributed by men (58.58% increase, APC 27.5%, 95% CI 2.4%-58.8% for urban men; 81.80% increase, APC 29.9%, 95% CI 4.0%-62.4% for eastern area men). Suicide was the third leading cause of injury among adolescents and young adults aged 10-24 years, accounting for 13.32% of all deaths caused by overall injuries, second only to traffic accidents (41.02%) and drowning (19.58%). There were significant differences in CMRs of suicide for both sexes within the disparate subgroups (Figure 5). The CMR of suicide increased with age, and the 20-24 years age group showed the greatest difference (RR 1.375, 95% CI 1.308-1.447), followed by the 10-14 years age group (RR 1.220, 95% CI 1.091-1.365) and the 15-19 years age group (RR 1.192, 95% CI 1.118-1.271). Although the CMR in rural regions was considerably higher than that in urban regions, the difference in the CMR between both sexes in urban regions (RR 1.297, 95% CI 1.204-1.397) indicated more significance than that between both sexes in the rural regions (RR 1.262, 95% CI 1.208-1.318). The CMR of suicide increased from the eastern area to the western area of China with statistically significant disparities, but the differences in CMR of suicide between men and women decreased from the eastern to western areas of China.

### Age-Specific and Cohort Trends of All-Cause Injury and Suicide During 2005-2019

**Figure 6:**
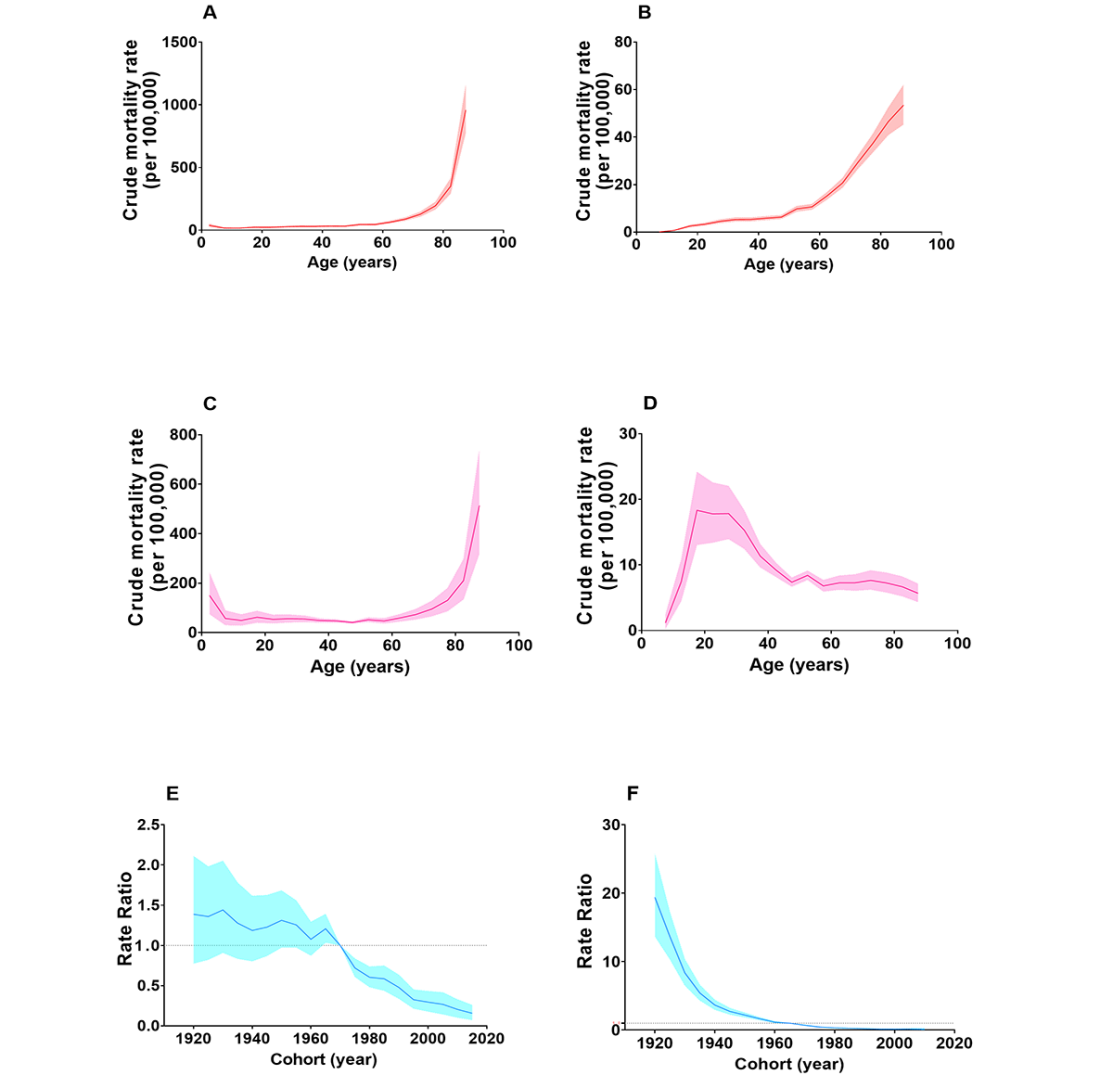
Age-specific and cohort trends of all-cause injury and suicide in China in 2005-2019. A. Cross-sectional age curve of all-cause injury among the 0-4 to 85+ years age group. B. Cross-sectional age curve of suicide among the 5-9 to 85+ years age group. C. Longitudinal age curve of all-cause injury among the 0-4 to 85+ years age group. D. Longitudinal age curve of suicide among the 5-9 to 85+ years group. E. The trend in the risk ratio of all-cause injury crude mortality rates among birth cohorts in 1920-1924 and 2015-2019 using the birth cohort of 1970-1974 as the reference group. F. The trend in the risk ratio of suicide crude mortality rates among the birth cohort in 1920-1924 and 2010-2014 by using the birth cohort of 1965-1969 as the reference group.

The trends of all-cause injury as well as age-specific suicide are demonstrated by cross-sectional age curves and longitudinal age curves, respectively (Figure 6). For all-cause injury, the pattern of the cross-sectional age change curves was identical to that of the longitudinal age curves, with 0-5 to 10-14 years age group showing a downward trend and ≥15 years group generally exhibiting a progressive increase with age, notably among older adults. For suicide, the age-specific cross-sectional suicide curve indicated that mortality rates increased with age, whereas the longitudinal age curve showed an inverted “U” skewed peak shape, with a gradual increase in the age groups from 5-9 to 15-19 years, reaching a maximum among the 15-19 to 25-29 years, a pronounced decrease with increasing age among the 25-29 to 45-49 years age groups, and a stable tendency over 50 years. Moreover, the net drift in suicide from 2005 to 2019 was more than SD 1% per year, at –5.75% per year (95% CI –6.42 to –5.28) (all age deviations=0, Wald *χ*^2^_15_=122.6; *P*<.001). Compared to that of suicide (99.77% decrease, AAPC –27.2%, 95% CI –30.1% to –24.1%), the cohort-related risk of all-cause injury (89.24% decrease, AAPC –11.1%, 95% CI –15.7% to –6.3%) exhibited a significant downward trend among the 1920-1924 to 2015-2019 birth cohorts (Figure 6).

### Temporal Trends of Burden in Injuries in China During 2005-2019

**Figure 7:**
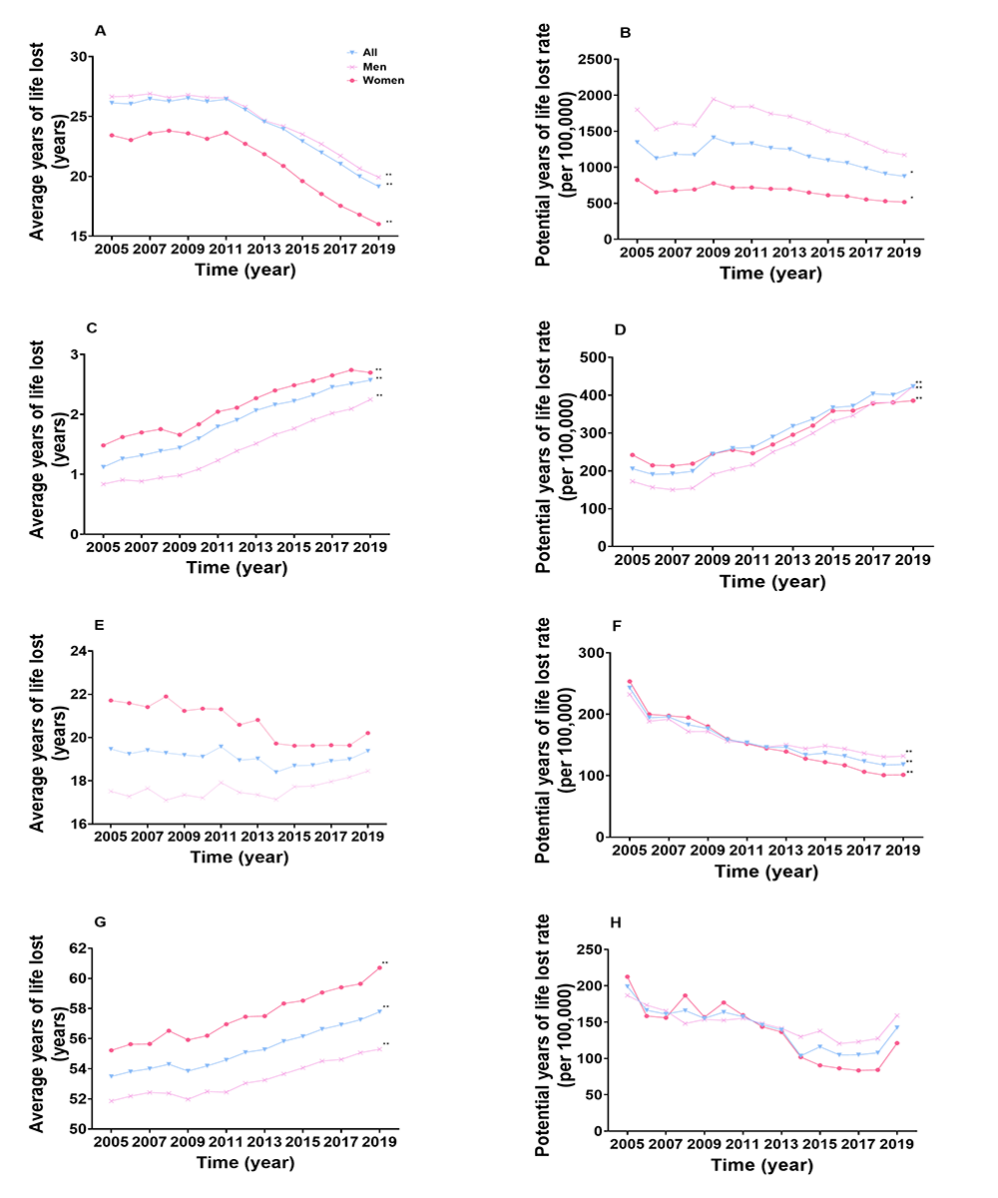
Trends in the average years of life lost and potential years of life lost rate in all-cause injury and suicide by sex and age in China in 2005-2019. A. The trend of average years of life lost in all-cause injury in the whole study population in 2005-2019. B. The trend of potential years of life lost rate in all-cause injury in the whole study population in 2005-2019. C. The trend of average years of life lost in all-cause injury among older adults in 2005-2019. D. The trend of potential years of life lost rate in all-cause injury among older adults in 2005-2019. E. The trend of average years of life lost in suicide in the study population in 2005-2019. F. The trend of potential years of life lost rate in suicide in the whole study population in 2005-2019. G. The trend of average years of life lost due to suicide among adolescents and young adults aged 10-24 years in 2005-2019. H. The trend of potential years of life lost rate in suicide among adolescents and young adults aged 10-24 years in 2005-2019. *P<.01, **P<.001 (for trends in 2005-2019).

In terms of all-cause injury in the whole population during 2005-2019, AYLL decreased by 26.71% (AAPC –2.2%, 95% CI –2.5% to –2.0%) and PYLLR decreased by 35.01% (AAPC –2.8%, 95% CI –4.7% to –0.8%), and both these trends were found to decrease faster in women (31.62% decrease, AAPC –2.7%, 95% CI –3.3% to –2.1% for AYLL; 37.29% decrease, AAPC –3.1%, 95% CI –5.1% to –1.0% for PYLLR) than in men (25.25% decrease, AAPC –2.1%, 95% CI –2.4% to –1.8% for AYLL; 34.93% decrease, AAPC –2.8%, 95% CI –5.6% to 0.1% for PYLLR) (Figure 7). For all-cause injury among older adults, AYLL (129.04% increase, AAPC 6.1%, 95% CI 5.4%-6.9%) and PYLLR (105.52% increase, AAPC 5.4%, 95% CI 2.4%-8.4%) increased consistently throughout the 2005-2019 period, with the same patterns occurring in both men (168.49% increase, AAPC 7.2%, 95% CI 6.1%-8.4% for AYLL; 145.85% increase, AAPC 6.6%, 95% CI 3.3%-9.9% for PYLLR) and women (81.81% increase, AAPC 4.2%, 95% CI 2.9%-5.6% for AYLL; 59.31% decrease, AAPC 4.1%, 95% CI 2.0%-6.2% for PYLLR). In the whole population, AYLL due to suicide, with higher AYLL among women, demonstrated an increasing tendency since 2014 (5.34% increase, APC 0.8%, 95% CI 0.5%-1.1%), especially for men (7.68% increase, APC 1.3%, 95% CI 0.9%-1.8%) (Figure 7). Distinctly, PYLLR of suicide in the whole population decreased by 51.34% (AAPC –4.9%, 95% CI –6.9% to –2.8%), while women (59.95% decrease, AAPC –6.2%, 95% CI –7.5% to –4.9%) showed a larger reduction than men (43.19% decrease, AAPC –3.7%, 95% CI –5.5% to –2.0%). For youths aged 10-24 years, a continuous upward trend occurred in AYLL in 2005-2019 (8.02% increase, AAPC 0.5%, 95% CI 0.4%-0.7%) similar to that among men, which increased by 6.63% (AAPC 0.5%, 95% CI 0.3%-0.7%) while that among women increased by 9.92% (AAPC 0.6%, 95% CI 0.4%-0.9%). PYLLR of suicide among adolescents and young adults aged 10-24 years remained stable in 2005-2019.

## Discussion

### Principal Findings

In this study, our main findings were that (1) the ASMR of all-cause injury decreased while the CMR remained unchanged in 2005-2019; (2) significant increases in ASMRs of all-cause injury among urban male older adults and western area female older adults were detected since 2013; (3) the ASMR of suicide among males aged 10-24 years in the whole population as well as among the urban and the eastern area residents reversed remarkably since 2017; (4) in the older adult group, AYLL and PYLL of all-cause injury showed a substantial increase in the past 15 years; and (5) in the 10-24 years age group, AYLL due to suicide showed a significantly increasing trend (Figure S6 of Multimedia Appendix 2). The slope of the decreasing trend of ASMR in men was smaller than that in women, which is in line with that reported in other studies [8,12]. This phenomenon may be caused by inequalities in the sex ratio at birth [13], personalities [14], coping manners, attitudes [15], diverse roles, identities in the society, pressure from different walks, possibilities, degrees of experiencing various hazards [8,15,16], and lifestyles [4]. Our study shows that the ASMR of injury in rural residents was higher than that in urban residents, which was consistent with that reported in some previous studies performed in low-income or high-income countries [5,17]. The western area in China had the highest ASMR among all the areas, which may be linked to the multiple environmental constructions, inequality in medical resources, enforcement of pertinent laws and regulations, and disparate prehospital treatments due to different economic levels in those locations [18].

The leading cause of injury death in mainland China is traffic accidents. The roads in rural regions and western areas of China are more rugged, coupled with low awareness of safety (less use of seat belts and helmets, drunk driving [19], breaking traffic laws, etc), lack of health resources, and incomplete road constructions and facilities, resulting in a significantly higher mortality rate than that in other places. Given China’s growing number of motor vehicles [20], effective policy development and stringent implementation such as the installation of a speed-limiting device [21] are particularly necessary. However, the slope decline in the rural regions was sharper than that in the urban regions, which might be due to the substantial resources and labor invested by the government [3] and comprehensive development that is being strengthened to bring about greater environmental transformations in rural regions.

With the aging of China’s society [22], it is imperative to minimize injury deaths of older adults. The factors for the fall-oriented reversal trend might be associated with physical health, psychological condition, environmental elements, and medical resources [23,24]. The physical capabilities of aging populations continue to deteriorate, and diseases such as osteoporosis, cardiovascular conditions, and weakened reactions may dramatically impair daily activities, obviously increasing the risk of injury. Owing to urbanization and economic development, the majority of the young people in rural regions migrate to urban regions, leaving the older adults alone (empty nests). Consequently, there is a lack of timely assistance when injuries from falls occur [25]. Meanwhile, as the older adults are typically eager to spend time with family, the absence of care will eventually lead to psychological illness, which might be another potential risk factor. The complexity of the terrain and poor public transportation, particularly in mountainous areas, increases the risk of falls among older adults, and the shortage of medical aid supplies or inefficient use of available resources prevents the older adults injured in falls from receiving timely and effective treatment [25], frequently increasing the possibility of death. Therefore, the government should allocate more funds to alleviate the environmental issues and lack of medical resources resulting from economic backwardness, improve medical technology to treat older adults’ illness more effectively, and pay more attention to older adults’ psychological health with more public welfare activities to bring comfort in their loneliness.

A considerable decline in the ASMR of overall suicide across all patterns of injuries was detected in our study, unlike that in the United States and several east-Asian countries with rapidly increasing tendency [26,27]. Consistent with other researches [28,29], our findings revealed that suicide death in men was significantly higher than that in women, which was lower than that reported in the United States [30] and some other Asian countries [31]. The role of women in the family has undergone a dramatic change in Chinese traditions, wherein women are required to stay at home to raise children. It is well known that the high risk for suicide is a consequence of individuals living in communities where there is lack of regular interpersonal contact and communication. Women now have more opportunities to leave the home to pursue their own goals and engage in more social activities, which relieves the psychological strain and helps reduce the possibility of suicidal thoughts. When confronted with stress, women are generally more motivated than men to communicate and release to alleviate the stress; thus, men have a greater potential to commit suicide than women under the same high-pressure environments. Moreover, women are keener to care about the quality of life [32,33], which invariably increases the sense of well-being and contributes to the decline in suicide mortality among women. Therefore, it is critical to pay more attention to men’s psychological health, relieve the societal pressure on men, enhance safety awareness education, and improve the overall satisfaction and quality of life. With a backward and undeveloped economy, more children in the family, and arduous and stressful life, many individuals in rural regions commit suicide to relieve themselves. However, as the economy of the country develops, people’s standards of living will rise, urbanization will continue to increase, more rural residents will relocate to urban regions, and their incomes will increase, which can result in a decline in the suicide rate.

Notably, the ASMR of suicide in the youth group aged 10-24 years has reversed in recent years, which is identical to the trend observed in Shanghai [34], and college students are reported to have higher risks of suicidality [35]. The uprising tendency in more high-income areas (the urban and eastern), especially among men, was significant, as reported in Japan [36]. Additionally, age patterns indicated that young people faced a higher suicide risk (when the net drift is more than or equal to SD 1% per year, it suggests that there is a more severe bias when the cross-sectional age trend is used to explain the natural history pattern rather than the longitudinal age curve to a great extent [11]). Economic development has pros and cons. With urbanization and economic growth’s positive effects on suicide steadily decreasing [37], competitive pressure has increased in all walks, and young people today undertake more stress in this fast-paced world, which is especially apparent among men in high-income regions [38]. On the one hand, young adults aged 20-24 years who have recently graduated and entered the society find it difficult to adjust to the pressures of the workplace and directly encounter economic issues, which differ from those in school. On the other hand, those who are studying are constantly under pressure from their peers and instructors. According to the interpersonal theory of suicide, perceived burdensomeness and a lack of belonging raise the potential of fatal suicide [39]. Consequently, youth resort to suicide deaths because they overlook their mental health and are not able to effectively manage their stress [40]. Based on the diathesis-stress model, vulnerability factors and stressful life events together increase the incidence of suicidality [41]. Due to the one-child policy adoption [42], the majority of the young Chinese generations are singletons, which increases the possibility of psychological strain since there are fewer adequate individuals with whom they can share when they run into difficulties. A concern that cannot be ignored is that the risk of suicidal ideation might also be worsened by an irregular lifestyle [43], including staying up too late and eating irregularly. Apart from the above, it is crucial to pay more attention to youths’ emotional issues as they are not capable of handling stress on their own, which might negatively affect their psychological well-being if they are not addressed properly. Further, a thwarted sensation of belonging may be caused by single parents or reconstituted families, which, along with the rising divorce rate in contemporary society, might leave certain psychological scars that could later result in negative emotional outbursts in youths. Therefore, surveillance procedures in dormitories and campuses should be reinforced so that youths who exhibit aberrant behavior can receive active psychological therapy and treatment. Additionally, mental health education classes and counseling services could be offered to improve advice on dealing with psychological strain. Moreover, the media and other platforms could be used to spread awareness about mental health to the public, which can reduce the negative forms of therapy for mental health issues resulting from shyness or narrow-mindedness and enable adolescents and young adults to develop the right concept of stress relief or positive psychotherapy. The burden of injury among youth has been increasing over years, which might be linked to the escalating mental health issues resulting from increased stress in the contemporary society. Youths are the cornerstone and the future of family and society; thus, AYLL of youth will contribute to a deeper negative impact on the social economy and structure. The government in China should be urged to pay greater attention to young people’s suicide as soon as possible in consideration of the sustained increase in AYLL.

### Limitations

Our study has several limitations. First, the DSP system had a high rate of missing data, which could cause bias in the mortality rate computation. Second, the increase in DSP from 161 in 2005 to 605 in 2013 could make the data skewed. Third, there was no in-depth study on the underlying causative factors of assorted patterns of injuries in this study, such as suicide, in which pesticide poisoning and hanging [44] were the major causes of death. One study [45] indicated that the major way to commit suicide in China has changed from pesticide poisoning and hanging to falls and jumping from heights. The risk factors for falls vary among older adults, for example, some older adults are more likely to fall due to an underlying disease while others may have a physical disability. Fourth, further investigations on the before-and-after comparison of injury mortality rates in different locations and age groups could not be performed after the government and key departments had implemented corresponding preventative and solution measures to investigate appropriate policies and strategies. Fifth, our study primarily addresses the loss of life-years due to premature mortality without addressing the loss of life-years owing to health loss from nonfatal injuries. Sixth, the trend of all-cause injury mortality varied largely in 2005-2009, which mainly resulted from underreporting of deaths (the entirety of the nationwide crude mortality underreporting rate was 16.68% [6271/37,603], with a weighted adjusted underreporting rate of 17.44% [46]), changing in reporting methods (from filling out case report cards to reported online), and external factors (such as epidemics, natural disasters, or socioeconomic events, especially the 2008 Wenchuan earthquake that resulted in 18,000 people missing [5]).

### Conclusion

Despite a markedly declining trend from 2005 to 2019, injury still accounts for a major part of deaths in China. The ASMR of suicide in adolescents and young adults has significantly reversed recently, while the ASMR of falls among older adults has increased since 2007. Our results provide an up-to-date scientific foundation for the establishment of policies and initiatives to prevent mortality due to cause-specific injury.

## Appendix

Multimedia Appendix 1 Formulae and methods of injury-related indices of mortality and burden.

Multimedia Appendix 2 Supplementary data on deaths and burdens of cause-specific injury.

## Abbreviations

AAPC: average annual percentage change
APC: annual percentage change
ASMR: age-standardized mortality rate
AYLL: average years of life lost
CDC: Centers for Disease Control and Prevention
CMR: crude mortality rate
DSP: disease surveillance points
PYLL: potential years of life lost
PYLLR: potential years of life lost rate
RR: risk ratio

## Data Availability

The data that support the findings of this study are available from the cause-of-death data set made by the Chinese Centers for Disease Control and Prevention, but some restrictions apply to the accessibility to the data.
