# Supplementary material for: Trends in Cause-Specific Injury Mortality in China in 2005-2019: Longitudinal Observational Study
Source: JMIR Public Health Surveill. 2023 Sep 15;9:e47902. doi: 10.2196/47902 (PMC10541646; doi:10.2196/47902)
Supplement: Multimedia Appendix 1 [file publichealth_v9i1e47902_app1.docx]

**Multimedia Appendix 1**

**Formulae and methods of injury-related indices on**

**mortality and burden**

- **Crude mortality rate (CMR)**

The crude mortality rates directly reflect the ratio of deaths caused by injury or a specific type of injury to the total population in a given location over a specific period, in terms of deaths per 100,000 population.

$$CMR=\frac{D}{N}\times100000/100000$$

*D* - *the number of deaths*; *N* - *the number of populations*

- **Age-standardized mortality rate (ASMR)**

The age-standardized rates are more suited for comparisons of various groups, which have eliminated disparities caused by different population sizes. Typically, calculations are performed using standardized demographic data. In the current study, the standardized population data were derived from China’s fifth census data [1] in 2000 using the analytical methods recommended by S. Rothman and K. Greenland [2]. We calculated also standardized rates in terms of deaths per 100,000 population.

- **Potential years of life lost (PYLL)**

Potential life years lost, also known as life expectancy lost due to death, is the aggregate of discrepancies between the expected life expectancy and the actual age at death for persons in a certain age group who die from a specific injury or all-cause injury. The reduction in life expectancy caused by injury mortality is further quantified using expected lifespan as a baseline, highlighting the negative impact of premature death on health. China's annual life expectancy values were extracted from World Bank data. Age groups that were above the life expectancy were not included.

$PYLL=\sum\left( L-\left( i+0.5 \right) \right)\times$*D_i_*

*L* - *life expectancy (years)*; *i* – the *median age of each age group*; *D_i_* - *the number of deaths at the age i*;

- **Average years of life lost (AYLL)**

Average life years lost is the average of discrepancies between the expected life expectancy and the actual age at death for persons in a certain age group who die from a specific injury or all-cause injury, which reflects the average loss in potential life years lost by each death as a result of a specific injury or all-cause injury.

$$AYLL=\frac{PYLL}{D}$$

*D* **-** *the whole number of* *deaths caused by* *a specific or all-cause injury during a certain time*.

- **Potential years of life lost rate (PYLLR)**

Potential years of life lost rate is the ratio of potential years of life lost to the overall population of the statistical group of a certain population during a certain period. It, a critical indicator of the population's level of health, is calculated using the mortality population's age distribution and is applied to show the level of risk associated with various populations and various causes of death.

$$PYLLR=\frac{PYLL}{N}\times100000/100000$$

*N* - *the whole number of* *a certain population during a certain time*.

**The average life expectancy in China, 2005-2019(years)**

| **Years** | **All** | **Male** | **Female** |
| --- | --- | --- | --- |
| *2005* | 73.0 | 71.3 | 74.8 |
| *2006* | 73.3 | 71.6 | 75.2 |
| *2007* | 73.6 | 71.8 | 75.5 |
| *2008* | 73.8 | 72.0 | 75.9 |
| *2009* | 74.1 | 72.2 | 76.2 |
| *2010* | 74.4 | 72.5 | 76.6 |
| *2011* | 74.7 | 72.7 | 76.9 |
| *2012* | 75.0 | 73.0 | 77.3 |
| *2013* | 75.3 | 73.2 | 77.6 |
| *2014* | 75.6 | 73.5 | 78.0 |
| *2015* | 75.9 | 73.8 | 78.3 |
| *2016* | 76.2 | 74.1 | 78.6 |
| *2017* | 76.5 | 74.3 | 78.8 |
| *2018* | 76.7 | 74.5 | 79.1 |
| *2019* | 76.9 | 74.8 | 79.2 |

**References**

1. Fan CC. Eurasian Geog Copyright © 2 Population Change and Regional Development in China: Insights Based on the 2000 Census.

2. G RS. Modern Epidemiology 3Ed. Lippincott Williams & Wilkins. 2008.
