# Supplementary material for: Trends in Cause-Specific Injury Mortality in China in 2005-2019: Longitudinal Observational Study
Source: JMIR Public Health Surveill. 2023 Sep 15;9:e47902. doi: 10.2196/47902 (PMC10541646; doi:10.2196/47902)

**Multimedia Appendix 2**

**Trends in Cause-Specific Injury Mortality in China in 2005-2019: Longitudinal Observational Study**

**Zixiang Ji*, BMed; Hengjing Wu*, PhD; Rongyu Zhu, BMed; Lu Wang, BSc; Yuzhu Wang, BMed; Lijuan Zhang, PhD**

Clinical Center for Intelligent Rehabilitation Research, Shanghai YangZhi Rehabilitation Hospital, Tongji University School of Medicine, Tongji University, Shanghai, China

*****these authors contributed equally

**Corresponding Author:**

Lijuan Zhang, PhD

Clinical Center for Intelligent Rehabilitation Research, Shanghai YangZhi Rehabilitation Hospital

Tongji University School of Medicine, Tongji University

50 Chifeng Road, Yangpu

Shanghai, 201619

China

Phone: 86 13817934887

Email: zhangxiaoyi@tongji.edu.cn

Table of Contents

**Table S11**

**Table S22**

**Table S34**

**Table S45**

**Table S56**

**Table S67**

**Table S78**

**Table S810**

**Figure S112**

**Figure S213**

**Figure S314**

**Figure S415**

**Figure S516**

**Figure S617**

**Table S1.** The International Classification of Diseases, 10th Revision (ICD-10) codes for types of injury

| Categories of injury death | ICD-10 |
| --- | --- |
| *****Injury, total***** | ***V01-Y89*** |
| Traffic accident | V01-V04, V06, V09-V80, V87, V89, V99 |
| Accidental poisoning | X40-X49 |
| Accidental falls | W00-W19 |
| Accidental fires | X00-X09 |
| Accidental drowning | W65-W74 |
| Other unintentional injuries | V05, V07-V08, V81-V86, V88, V90-V98, W20-W64, W75-W99, X10-X39, X50-X59, Y40- Y86, Y88, Y89 |
| Suicide | X60-X84, Y87.0 |
| Suicide by poisoning | X60-X69 |
| Suicide by hanging and strangulation | X70 |
| Suicide by drowning and submersion | X71 |
| Suicide by firearm discharge and explosive material | X72-X75 |
| Suicide by jumping | X80 |
| Other intentional injuries | X85-Y09, Y35, Y36, Y87.1 |

**Table S2.** The data on annual injury death and all-cause deaths in the population derived from the disease surveillance points system (DSP) collected by the Chinese Center for Disease Control and Prevention (CDC) between 2005 and 2019

| Year | Injury Death | | |  | All Death | | |  | Number of Population | | |
| --- | --- | --- | --- | --- | --- | --- | --- | --- | --- | --- | --- |
|  | **Man(%)** | **Woman(%)** | **Total** |  | **Man(%)** | **Woman(%)** | **Total** |  | **Man(%)** | **Woman(%)** | **Total** |
| 2005 | 24680(66.8%) | 12280(33.2%) | 36960 |  | 251926(57.6%) | 185564(42.4%) | 437490 |  | 36553090(51.1%) | 34934187(48.9%) | 71487277 |
| 2006 | 19315(67.8%) | 9183(32.2%) | 28498 |  | 202885(58.5%) | 144172(41.5%) | 347057 |  | 33707590(51.1%) | 32304648(48.9%) | 66012238 |
| 2007 | 21859(68.5%) | 10037(31.5%) | 31896 |  | 235792(58.8%) | 165216(41.2%) | 401008 |  | 36490345(51.1%) | 34986128(48.9%) | 71476473 |
| 2008 | 22506(68.2%) | 10511(31.8%) | 33017 |  | 251137(59.1%) | 173546(40.9%) | 424683 |  | 37759875(51.1%) | 36168624(48.9%) | 73928499 |
| 2009 | 27807(69.6%) | 12122(30.4%) | 39929 |  | 258482(59.1%) | 179068(40.9%) | 437550 |  | 38286132.85(51.0%) | 36734355.65(49.0%) | 75020488.5 |
| 2010 | 27764(69.9%) | 11978(30.1%) | 39742 |  | 268269(59.2%) | 184942(40.8%) | 453211 |  | 40176531(51.0%) | 38590095(49.0%) | 78766626 |
| 2011 | 27351(70.3%) | 11576(29.7%) | 38927 |  | 264702(59.4%) | 180775(40.6%) | 445477 |  | 39380622(50.9%) | 38015856(49.1%) | 77396478 |
| 2012 | 26607(69.5%) | 11683(30.5%) | 38290 |  | 271317(59.0%) | 188519(41.0%) | 459836 |  | 39344367(51.0%) | 37871630(49.0%) | 77215997 |
| 2013 | 80044(69.2%) | 35563(30.8%) | 115607 |  | 859299(58.7%) | 604552(41.3%) | 1463851 |  | 115772192(50.9%) | 111464092(49.1%) | 227236284 |
| 2014 | 86523(69.1%) | 38642(30.9%) | 125165 |  | 965261(58.7%) | 678116(41.3%) | 1643377 |  | 129341238(51.0%) | 124269657(49.0%) | 253610895 |
| 2015 | 83608(67.9%) | 39587(32.1%) | 123195 |  | 973713(58.2%) | 699532(41.8%) | 1673245 |  | 130792727(50.8%) | 126778650(49.2%) | 257571377 |
| 2016 | 85857(67.2%) | 41939(32.8%) | 127796 |  | 1011826(58.0%) | 731715(42.0%) | 1743541 |  | 134787443(50.9%) | 129985425(49.1%) | 264772868 |
| 2017 | 84846(66.9%) | 42048(33.1%) | 126894 |  | 1035486(58.1%) | 747969(41.9%) | 1783455 |  | 137797213(50.8%) | 133338458(49.2%) | 271135671 |
| 2018 | 81888(66.0%) | 42133(34.0%) | 124021 |  | 1055869(57.9%) | 766661(42.1%) | 1822530 |  | 138495174(50.9%) | 133759675(49.1%) | 272254849 |
| 2019 | 82627(65.3%) | 43940(34.7%) | 126567 |  | 1081860(57.9%) | 785664(42.1%) | 1867524 |  | 140579648(50.8%) | 136293497(49.2%) | 276873145 |

**Table S3.** The data on crude mortality rate (CMR) and 95% confidence interval (*95%CI*)^a^ of the six leading causes of injury between men and women by region and area in China from 2005 to 2019

| Types of Injury |  |  | Region | |  | Area | | |  | Subtotal | Total |
| --- | --- | --- | --- | --- | --- | --- | --- | --- | --- | --- | --- |
|  |  |  | **Urban** | **Rural** |  | **Eastern** | **Central** | **Western** |  |  |  |
| Traffic accident | Man |  | 17.56  (17.43-17.69) | 27.92  (27.80-8.03) |  | 23.14  (23.01-23.28) | 24.52  (24.37-24.67) | 26.28  (26.10-26.46) |  | 24.45  (24.35-24.52) | 16.57  (16.52-16.63) |
|  | Woman |  | 6.75  (6.67-6.83) | 9.41  (9.35-9.48) |  | 8.31  (8.22-8.39) | 8.70  (8.61-8.79) | 8.42  (8.32-8.53) |  | 8.51  (8.46-8.56) |  |
| Poisoning | Man |  | 3.04  (2.98-3.09) | 4.82  (4.77-4.87) |  | 3.00  (2.90-3.05) | 3.97  (3.91-4.03) | 6.36  (6.28-6.45) |  | 4.22  (4.18-4.26) | 3.00  (2.98-3.02) |
|  | Woman |  | 1.29  (1.26-1.33) | 1.98  (1.95-2.01) |  | 1.42  (1.39-1.46) | 1.79  (1.75-1.83) | 2.12  (2.07-2.17) |  | 1.75  (1.72-1.77) |  |

| Falls | Man |  | 9.98  (9.88-10.08) | 12.33  (12.25-12.40) |  | 11.01  (10.91-11.10) | 9.74  (9.65-9.83) | 14.74  (14.60-14.87) |  | 11.54  (11.48-11.60) | 9.66  (9.62-9.70) |
| --- | --- | --- | --- | --- | --- | --- | --- | --- | --- | --- | --- |
|  | Woman |  | 7.33  (7.24-7.41) | 7.99  (7.93-8.05) |  | 9.67  (9.58-9.76) | 5.23  (5.16-5.30) | 8.18  (8.08-8.28) |  | 7.76  (7.71-7.81) |  |
| Fires | Man |  | 0.59  (0.57-0.61) | 0.97  (0.95-0.99) |  | 0.84  (0.81-0.86) | 0.91  (0.89-0.94) | 0.75  (0.72-0.78) |  | 0.90  (0.88-0.91) | 0.64  (0.63-0.65) |
|  | Woman |  | 0.27  (0.26-0.29) | 0.51  (0.49-0.52) |  | 0.42  (0.41-0.44) | 0.47  (0.44-0.49) | 0.37  (0.35-0.40) |  | 0.43  (0.42-0.44) |  |
| Drowning | Man |  | 3.17  (3.11-3.22) | 5.77  (5.72-5.82) |  | 3.96  (3.90-4.01) | 4.92  (4.85-4.99) | 6.25  (6.16-6.34) |  | 4.89  (4.85-4.93) | 3.75  (3.72-3.77) |
|  | Woman |  | 1.73  (1.69-1.77) | 3.01  (2.98-3.05) |  | 2.11  (2.06-2.15) | 2.64  (2.59-2.69) | 3.16  (3.10-3.22) |  | 2.58  (2.55-2.61) |  |
| Suicide | Man |  | 5.73  (5.60-5.85) | 9.80  (9.73-9.87) |  | 7.37  (7.30-7.45) | 9.97  (9.87-10.06) | 7.96  (7.87-8.06) |  | 8.43  (8.38-8.48) | 7.46  (7.43-7.50) |
|  | Woman |  | 4.32  (4.26-4.38) | 7.62  (7.56-7.68) |  | 5.63  (5.56-5.69) | 8.04  (7.95-8.12) | 5.64  (5.56-5.73) |  | 6.50  (6.45-6.54) |  |

^a^ CMR and *95%CI* per 100,000 for diverse types of injury

**Table S4.** The data on age-standardized mortality rate (ASMR) and 95% confidence interval (*95%CI*)^a^ of the six leading causes of injury between men and women by region and area in China from 2005 to 2019

| Types of Injury |  | Region | |  | Area | | |  | Subtotal | Total |
| --- | --- | --- | --- | --- | --- | --- | --- | --- | --- | --- |
|  |  | **Urban** | **Rural** |  | **Eastern** | **Central** | **Western** |  |  |  |

| Traffic accident | Man | 14.90  (14.78-15.01) | 25.15  (25.04-25.26) |  | 19.32  (19.19-19.44) | 21.68  (21.55-21.82) | 24.74  (24.56-24.91) |  | 21.57  (21.48-21.65) | 14.33  (14.28-14.37) |
| --- | --- | --- | --- | --- | --- | --- | --- | --- | --- | --- |
|  | Woman | 5.43  (5.36-5.50) | 7.84  (7.78-7.90) |  | 6.40  (6.33-6.47) | 7.25  (7.17-7.33) | 7.44  (7.35-7.54) |  | 7.00  (6.95-7.04) |  |
| Poisoning | Man | 2.50  (2.46-2.55) | 4.16  (4.11-4.20) |  | 2.40  (2.36-2.44) | 3.36  (3.31-3.42) | 5.71  (5.63-5.79) |  | 3.58  (3.55-3.61) | 2.49  (2.47-2.51) |
|  | Woman | 1.02  (0.99-1.05) | 1.60  (1.57-1.63) |  | 1.04  (1.01-1.06) | 1.44  (1.40-1.47) | 1.88  (1.83-1.93) |  | 1.40  (1.38-1.42) |  |
| Falls | Man | 7.29  (7.22-7.36) | 9.62  (9.55-9.68) |  | 7.47  (7.40-7.54) | 7.80  (7.72-7.88) | 12.13  (12.02-12.25) |  | 8.80  (8.75-8.85) | 6.51  (6.48-6.54) |
|  | Woman | 3.87  (3.82-3.92) | 4.33  (4.29-4.36) |  | 4.15  (4.10-4.19) | 3.18  (3.13-3.22) | 5.33  (5.26-5.40) |  | 4.16  (4.13-4.19) |  |
| Fires | Man | 0.45  (0.43-0.47) | 0.76  (0.75-0.78) |  | 0.59  (0.57-0.61) | 0.75  (0.72-0.77) | 0.63  (0.60-0.65) |  | 0.69  (0.68-0.70) | 0.46  (0.45-0.47) |
|  | Woman | 0.18  (0.17-0.19) | 0.34  (0.33-0.35) |  | 0.24  (0.23-0.25) | 0.33  (0.31-0.35) | 0.28  (0.26-0.30) |  | 0.28  (0.27-0.29) |  |
| Drowning | Man | 3.26  (3.20-3.32) | 5.72  (5.67-5.78) |  | 3.90  (3.84-3.96) | 4.96  (4.89-5.03) | 6.31  (6.22-6.40) |  | 4.93  (4.89-4.97) | 3.60  (3.58-3.63) |
|  | Woman | 1.53  (1.49-1.57) | 2.56  (2.52-2.59) |  | 1.66  (1.62-1.69) | 2.27  (2.23-2.32) | 2.95  (2.89-3.02) |  | 2.22  (2.20-2.25) |  |
| Suicide | Man | 4.55  (4.49-4.61) | 7.99  (7.93-8.05) |  | 5.69  (5.63-5.75) | 8.00  (7.92-8.08) | 6.96  (6.87-7.05) |  | 6.79  (6.75-7.83) | 5.83  (5.81-5.86) |
|  | Woman | 3.27  (3.21-3.32) | 5.82  (5.77-5.87) |  | 4.05  (4.00-4.10) | 6.00  (5.93-6.07) | 4.84  (4.76-4.91) |  | 4.92  (4.88-4.96) |  |

^a^ ASMR and *95%CI* per 100,000 for diverse types of injury

**Table S5.** The data on Risk Ratio (RR) values and 95% confidence interval (*95%CI*) for CMR of all-cause injury and the six leading causes of injury by sex, region, and area in China from 2005 to 2019

| Types of Injury | Sex |  | Region |  | Area | |
| --- | --- | --- | --- | --- | --- | --- |
|  | **Man/Woman** |  | **Rural/Urban** |  | **Western/Eastern** | **Western/Central** |
| Traffic accident | 2.871 (2.851-2.892) |  | 1.542 (1.531-1.553) |  | 1.112 (1.104-1.121) | 1.051 (1.043-1.059) |
| Poisoning | 2.418 (2.379-2.457) |  | 1.586 (1.559-1.613) |  | 1.939 (1.904-1.974) | 1.488 (1.463-1.514) |
| Falls | 1.486 (1.474-1.499) |  | 1.176  (1.166-1.187) |  | 1.120 (1.109-1.130) | 1.539 (1.523-1.556) |
| Fires | 2.100 (2.032-2.172) |  | 1.712 (1.649-1.778) |  | 0.894  (0.858-0.932) | 0.816  (0.783-0.851) |
| Drowning | 1.897 (1.872-1.924) |  | 1.793 (1.766-1.822) |  | 1.564  (1.539-1.590) | 1.253  (1.234-1.273) |
| Suicide | 1.298 (1.286-1.310) |  | 1.738 (1.719-1.757) |  | 1.055 (1.042-1.068) | 0.762 (0.753-0.771) |
| All-cause Injury | 2.024 (2.016-2.032) |  | 1.492 (1.486-1.499) |  | 1.179 (1.174-1.185) | 1.146 (1.140-1.151) |

**Table 6.** The data on Risk Ratio (RR) values and 95% confidence interval (*95%CI*) for ASMR of all-cause injury and the six leading causes of injury by sex, region, and area in China from 2005 to 2019

| Types of Injury | Sex |  | Region |  | Area | |
| --- | --- | --- | --- | --- | --- | --- |
|  | **Man/Woman** |  | **Rural/Urban** |  | **Western/Eastern** | **Western/Central** |
| Traffic accident | 3.082 (3.058-3.105) |  | 1.627 (1.615-1.639) |  | 1.263 (1.253-1.274) | 1.120 (1.111-1.130) |
| Poisoning | 2.555 (2.512-2.600) |  | 1.658  (1.629-1.688) |  | 2.230 (2.188-2.273) | 1.597 (1.568-1.626) |
| Falls | 2.113 (2.094-2.132) |  | 1.251  (1.239-1.263) |  | 1.506 (1.491-1.521) | 1.597 (1.580-1.615) |
| Fires | 2.445 (2.359-2.535) |  | 1.750 (1.681-1.822) |  | 1.109  (1.061-1.159) | 0.848  (0.811-0.885) |
| Drowning | 2.217 (2.184-2.250) |  | 1.731 (1.702-1.761) |  | 1.669  (1.639-1.698) | 1.280 (1.259-1.302) |
| Suicide | 1.380 (1.367-1.394) |  | 1.767 (1.747-1.788) |  | 1.220 (1.205-1.236) | 0.849 (0.838-0.859) |
| All-cause Injury | 2.397 (2.387-2.407) |  | 1.577 (1.570-1.584) |  | 1.408 (1.401-1.415) | 1.214 (1.208-1.219) |

**Table S7.** The data on the trend in age-standardized mortality rate (ASMR) per 100,000 of injury by sex, region, area, age, and injury type in China, 2005-2019

| Group | Trend 1 | | |  | Trend 2 | | | Trend 3 | | |  | AAPC(*95%CI*) | *P* |
| --- | --- | --- | --- | --- | --- | --- | --- | --- | --- | --- | --- | --- | --- |
|  | **Year** | **APC(*95%CI*)** | ***P*** |  | **year** | **APC(*95%CI*)** | ***P*** | **year** | **APC(*95%CI*)** | ***P*** |  |  |  |
| Sex |  |  |  |  |  |  |  |  |  |  |  |  |  |
| Man | 2005-2007 | -7.3(-15.4 to 1.6) | .09 |  | 2007-2010 | 7.3(-1.6 to 17.0) | .10 | 2010-2019 | -4.3(-4.8 to -3.8) | <.001 |  | -2.4(-4.2 to -0.5) | .01 |
| Woman | 2005-2007 | -10.3(-15.9 to -4.2) | .006 |  | 2007-2010 | 3.0(-3.4 to 9.8) | .31 | 2010-2019 | -3.4(-3.8 to -3.0) | <.001 |  | -3.1(-4.4 to -1.7) | <.001 |
| Region |  |  |  |  |  |  |  |  |  |  |  |  |  |
| Urban | 2005-2007 | -5.8(-32.8 to 32.1) | .69 |  | 2007-2015 | -0.4(-3.7 to 3.1) | .81 | 2015-2019 | -4.5(-9.8 to 1.1) | .10 |  | -2.3(-6.7 to 2.2) | .30 |
| Rural | 2005-2007 | -7.3(-18.5 to 5.4) | .21 |  | 2007-2010 | 6.7(-5.7 to 20.8) | .25 | 2010-2019 | -4.8(-5.5 to -4.0) | <.001 |  | -2.8(-5.4 to -0.1) | .04 |
| Area |  |  |  |  |  |  |  |  |  |  |  |  |  |
| Eastern | 2005-2007 | -8.6(-19.3 to 3.5) | .13 |  | 2007-2010 | 6.2(-5.5 to 19.5) | .26 | 2010-2019 | -3.8(-4.5 to -3.0) | <.001 |  | -2.4(-4.9 to 0.1) | .06 |
| Central | 2005-2007 | -7.6(-14.4 to -0.3) | .04 |  | 2007-2010 | 3.2(-4.4 to 11.4) | .37 | 2010-2019 | -4.3(-4.8 to -3.9) | <.001 |  | -3.3(-4.8 to -1.6) | <.001 |
| Western | 2005-2007 | -8.4(-17.1 to 1.2) | .08 |  | 2007-2010 | 9.1(-0.4 to 19.6) | .06 | 2010-2019 | -4.0(-4.5 to -3.4) | <.001 |  | -2.0(-3.9 to 0.0) | .06 |
| Age |  |  |  |  |  |  |  |  |  |  |  |  |  |
| ≤39 | 2005-2007 | -7.1(-23.5 to 12.7) | .40 |  | 2007-2011 | 4.8(-4.8 to 15.5) | .29 | 2011-2019 | -7.5(-9.0 to -6.0) | <.001 |  | -4.1(-7.2 to -0.8) | .02 |
| 40-64 | 2005-2011 | 0.2(-3.2 to 3.6) | .91 |  | 2011-2017 | -2.2(-5.0 to 0.6) | .10 | 2017-2019 | -7.5(-17.0 to 3.1) | .13 |  | -7.5(-17.0 to 3.1) | .13 |
| ≥65 | 2005-2010 | 0.7(-6.6 to 8.6) | .83 |  | 2010-2013 | -4.4(-31.2 to 32.8) | .75 | 2013-2019 | 1.0(-1.6 to 3.7) | .39 |  | -0.3(-6.4 to 6.2) | .93 |
| Injury types |  |  |  |  |  |  |  |  |  |  |  |  |  |
| Traffic accident | 2005-2007 | -5.0(-22.4 to 16.3) | .57 |  | 2007-2010 | 15.4(-3.6 to 38.2) | .10 | 2010-2019 | -5.7(-6.8 to -4.6) | <.001 |  | -1.4(-5.3 to 2.6) | .49 |
| Poisoning | 2005-2007 | -7.8(-21.2 to 7.7) | .26 |  | 2007-2015 | 0.0(-1.6 to 1.6) | .99 | 2015-2019 | -6.0(-8.5 to -3.3) | .001 |  | -2.9(-4.9 to -0.8) | .007 |
| Falls | 2005-2010 | -0.7(-3.6 to 2.2) | .57 |  | 2010-2013 | -3.1(-15.0 to 10.5) | .59 | 2013-2019 | 3.5(2.4-4.6) | <.001 |  | 0.5(-2.0 to 3.1) | .67 |
| Fires | 2005-2013 | -4.0(-6.9 to -1.0) | .02 |  | 2013-2016 | -9.2(-23.8 to 8.3) | .24 | 2016-2019 | 1.6(-6.9 to 10.8) | .68 |  | -4.0(-7.5 to -0.3) | .04 |
| Drowning | 2005-2007 | -8.1(-25.5 to 13.4) | .37 |  | 2007-2016 | -2.7(-4.6 to -0.8) | .01 | 2016-2019 | -7.7(-14.0 to -0.9) | .03 |  | -4.6(-7.4 to -1.7) | .002 |
| Suicide | 2005-2007 | -11.1(-18.7 to -2.9) | .02 |  | 2007-2011 | -6.9(-11.2 to -2.3) | .01 | 2011-2019 | -4.8(-5.6 to -4.1) | <.001 |  | -6.3(-7.8 to -4.9) | <.001 |

APC: annual percentage change, shows the change rate for a certain segment within 2005-2019; AAPC: average annual percentage change, shows the average change rate during 2005-2019; *95%CI*: 95% confidence interval

**Table S8.** The data on the trend in age-standardized mortality rate (ASMR) per 100,000 of suicide by sex, region, area, and crude mortality rate (CMR) per 100,000 by age in China, 2005-2019

| Group | Trend 1 | | |  | Trend 2 | | | Trend 3 | | |  | AAPC(*95%CI*) | *P* |
| --- | --- | --- | --- | --- | --- | --- | --- | --- | --- | --- | --- | --- | --- |
|  | **Year** | **APC(*95%CI*)** | ***P*** |  | **year** | **APC(*95%CI*)** | ***P*** | **year** | **APC(*95%CI*)** | ***P*** |  |  |  |
| Sex |  |  |  |  |  |  |  |  |  |  |  |  |  |
| Man | 2005-2012 | -7.5(-9.3 to -5.7) | <.001 |  | 2012-2015 | -1.5(-10.1 to 8.0) | .72 | 2015-2019 | -4.9(-7.7 to -2.1) | .005 |  | -5.5(-7.3 to -3.7) | <.001 |
| Woman | 2005-2007 | -11.3(-17.8 to -4.4) | .007 |  | 2007-2011 | -7.3(-11.1 to -3.5) | .003 | 2011-2019 | -6.4(-7.0 to -5.7) | <.001 |  | -7.4(-8.6 to -6.1) | <.001 |
| Region |  |  |  |  |  |  |  |  |  |  |  |  |  |
| Urban | 2005-2011 | -9.0(-12.3 to -5.4) | .001 |  | 2011-2015 | -0.3(-9.6 to 10.1) | .95 | 2015-2019 | -5.4(-9.9 to -0.7) | .03 |  | -5.5(-8.3 to -2.7) | <.001 |
| Rural | 2005-2007 | -9.0(-13.8 to -3.9) | .005 |  | 2007-2013 | -7.2(-8.5 to -5.9) | <.001 | 2013-2019 | -5.2(-5.8 to -4.6) | <.001 |  | -6.6(-7.4 to -5.8) | <.001 |
| Area |  |  |  |  |  |  |  |  |  |  |  |  |  |
| Eastern | 2005-2007 | -12.9(-23.5 to -0.8) | .04 |  | 2007-2011 | -6.8(-13.2 to 0.1) | .05 | 2011-2019 | -4.1(-5.2 to -3.0) | <.001 |  | -6.2(-8.4 to -3.9) | <.001 |
| Central | 2005-2012 | -8.4(-9.7 to -7.0) | <.001 |  | 2012-2015 | -3.9(-10.7 to 3.5) | .25 | 2015-2019 | -6.7(-9.0 to -4.4) | <.001 |  | -7.0(-8.4 to -5.5) | <.001 |
| Western | 2005-2012 | -7.2(-10.4 to -4.0) | .001 |  | 2012-2016 | -2.2(-10.4 to 6.7) | .56 | 2016-2019 | -6.5(-14.3 to 2.0) | .11 |  | -5.7(-8.4 to -2.8) | <.001 |
| Age^a^ |  |  |  |  |  |  |  |  |  |  |  |  |  |
| 10- | 2005-2011 | 0.9(-7.1 to 9.5) | .81 |  | 2011-2014 | -11.2(-42.7 to 37.6) | .54 | 2014-2019 | 8.8(1.6 to 16.5) | .02 |  | 0.8(-7.4 to 9.8) | .85 |
| 15- | 2005-2009 | -8.4(-13.6 to -2.8) | .01 |  | 2009-2017 | -3.7(-6.1 to -1.2) | .009 | 2017-2019 | 15.1(-0.4 to 33.0) | .06 |  | -2.6(-5.0 to -0.1) | .04 |
| 20- | 2005-2011 | -5.6(-9.5 to -1.5) | .02 |  | 2011-2017 | -11.9(-16.1 to -7.5) | <.001 | 2017-2019 | 28.4(5.5 to 56.2) | .02 |  | -4.2(-7.3 to -1.1) | .009 |
| 25- | 2005-2007 | -13.1(-38.2 to 22.2) | .36 |  | 2007-2016 | -1.6(-4.8 to 1.6) | .27 | 2016-2019 | -7.4(-17.6 to 3.9) | .16 |  | -4.6(-9.1 to 0.1) | .06 |
| 30- | 2005-2007 | -17.9(-37.3 to 7.6) | .13 |  | 2007-2017 | -2.7(-5.1 to -0.2) | .04 | 2017-2019 | -8.3(-26.4 to 14.3) | .38 |  | -5.8(-9.8 to -1.6) | .008 |
| 35- | 2005-2010 | -11.6(-15.2 to -7.8) | <.001 |  | 2010-2017 | -7.4(-10.4 to -4.4) | .001 | 2017-2019 | -2.4(-18.0 to 16.3) | .76 |  | -8.2(-10.7 to -5.7) | <.001 |
| 40- | 2005-2011 | -9.9(-13.5 to -6.2) | <.001 |  | 2011-2015 | -4.6(-14.0 to 5.8) | .32 | 2015-2019 | -11.7(-16.8 to -6.3) | .002 |  | -8.9(-11.8 to -6.0) | <.001 |
| 45- | 2005-2007 | -13.1(-33.0 to 12.7) | .24 |  | 2007-2013 | -5.8(-11.4 to 0.1) | .05 | 2013-2019 | -4.8(-7.3 to -2.2) | .004 |  | -6.5(-10.0 to -2.8) | .001 |
| 50- | 2005-2011 | -12.1(-20.8 to -7.6) | <.001 |  | 2011-2017 | 5.2(2.0 to 12.8) | .02 | 2017-2019 | -22.7(-30.6 to -13.1) | .004 |  | -6.8(-8.1 to -5.4) | <.001 |
| 55- | 2005-2010 | -10.1(-13.0 to -7.2) | <.001 |  | 2010-2017 | -7.0(-8.8 to -5.2) | <.001 | 2017-2019 | 2.7(-6.9 to 13.3) | .54 |  | -6.8(-8.4 to -5.2) | <.001 |
| 60- | 2005-2012 | -8.1(-17.9 to -5.1) | <.001 |  | 2012-2017 | 1.2(-1.4 to 7.8) | .35 | 2017-2109 | -15.7(-23.5 to -8.6) | .003 |  | -6.0(-7.2 to -4.8) | <.001 |
| 65- | 2005-2009 | -11.3(-21.8 to -5.7) | .004 |  | 2009-2017 | -1.3(-2.3 to 5.7) | .26 | 2017-2019 | -14.5(-21.1 to -7.5) | .02 |  | -6.2(-7.1 to -4.9) | <.001 |
| 70- | 2005-2011 | -9.8(-13.0 to -6.5) | <.001 |  | 2011-2014 | -1.8(-19.6 to 20.0) | .84 | 2014-2019 | -6.8(-9.7 to -3.9) | .001 |  | -7.1(-10.6 to -3.4) | .001 |
| 75- | 2005-2007 | -15.0(-28.1 to 0.5) | .06 |  | 2007-2017 | -7.4(-8.5 to -6.2) | <.001 | 2017-2019 | -4.0(-14.3 to 7.5) | .42 |  | -8.0(-10.3 to -5.7) | <.001 |
| 80- | 2005-2010 | -7.4(-11.3 to -3.4) | .004 |  | 2010-2013 | -11.4(-27.9 to 8.8) | .21 | 2013-2019 | -8.3(-10.2 to -6.4) | <.001 |  | -8.7(-12.2 to -5.0) | <.001 |
| 85- | 2005-2010 | 11.5(-7.4 to 72.4) | .12 |  | 2010-2013 | -27.0(-35.1 to -0.7) | .04 | 2013-2019 | -5.8(-22.9 to 17.9) | .67 |  | -5.3(-8.4 to 1.3) | .07 |

^a^ Because of low levels in the age groups 0-4 and 5-9 years old, annual trends of ASMRs in suicide could not be calculated and were not shown in the table. APC: annual percentage change, shows the change rate for a certain segment within 2005-2019; AAPC: average annual percentage change, shows the average change rate during 2005-2019; *95%CI*: 95% confidence interval

**Figure S1.** The rank of crude mortality rate (CMR) and age-standardized mortality rate (ASMR) per 100,000 for diverse types of injury of injury types by region (urban, rural) for the whole population derived from the disease surveillance points system (DSP) collected by the Chinese Center for Disease Control and Prevention (CDC) between 2005 and 2019

**
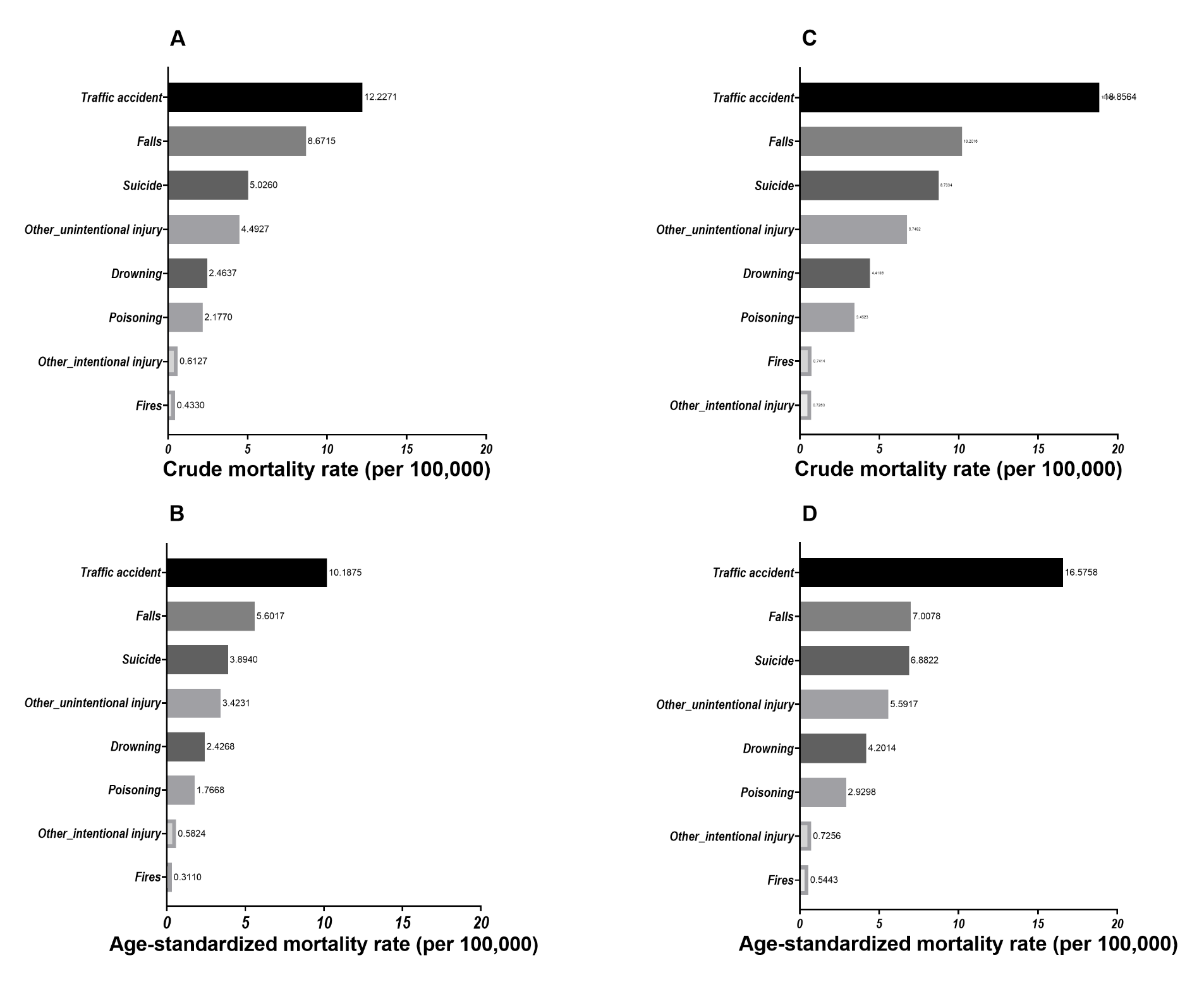
**

1. Crude mortality rates in the urban regions. B. Age-standardized mortality rates in urban regions. C. Crude mortality rates in the rural regions. D. Age-standardized mortality rates in rural regions.

**Figure S2.** The rank of crude mortality rate (CMR) and age-standardized mortality rate (ASMR per 100,000 of injury types by area (eastern, central, western)) for the whole population derived from the disease surveillance points system (DSP) collected by the Chinese Center for Disease Control and Prevention (CDC) between 2005 and 2019


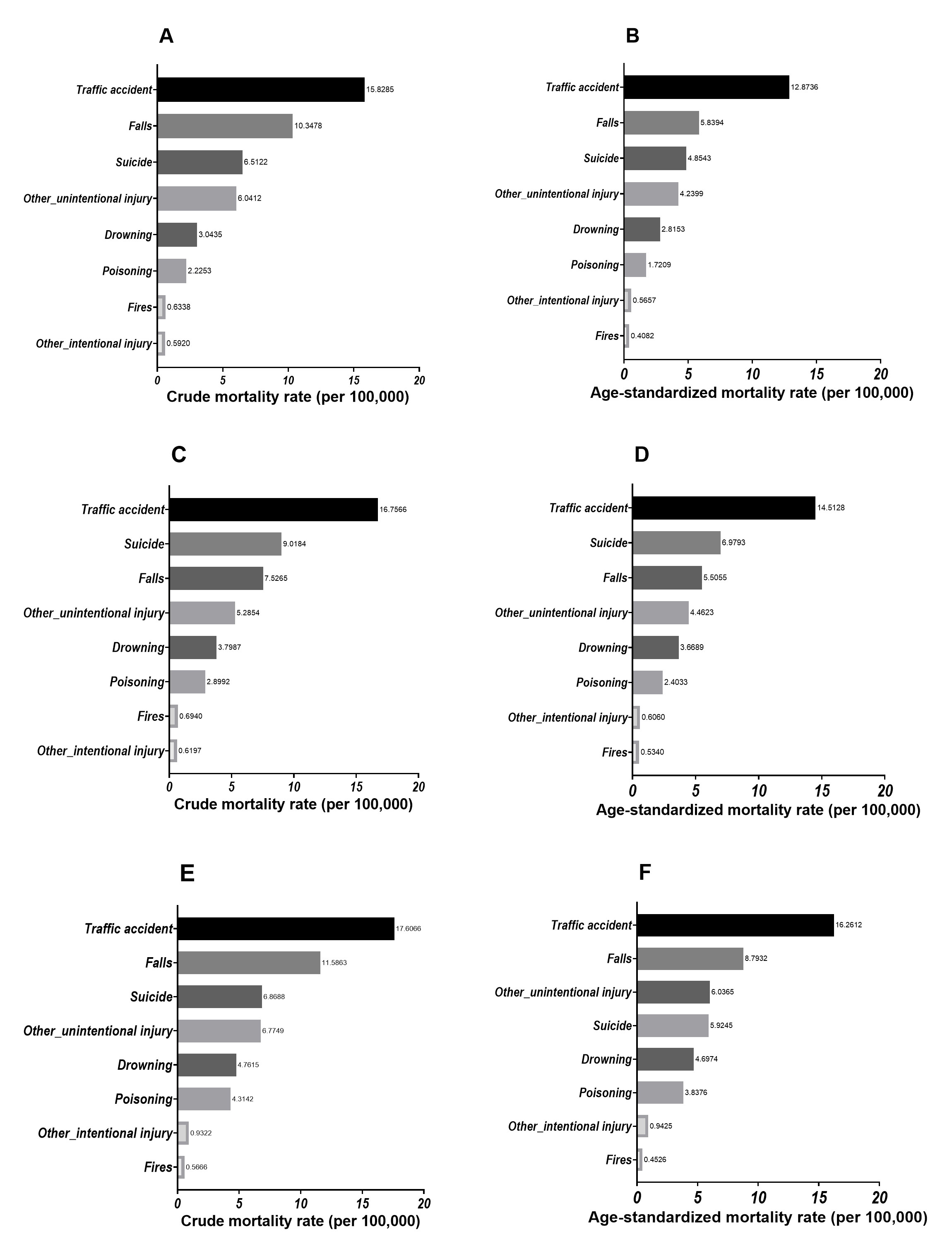


1. Crude mortality rates in the eastern. B. Age-standardized mortality rates in the eastern. C. Crude mortality rates in the central. D. Age-standardized mortality rates in the central. E. Crude mortality rates in the western. F. Age-standardized mortality rates in the western.

**Figure S3.** The trend of disparate types of injuries among people aged 65+ years in China, 2005-2019


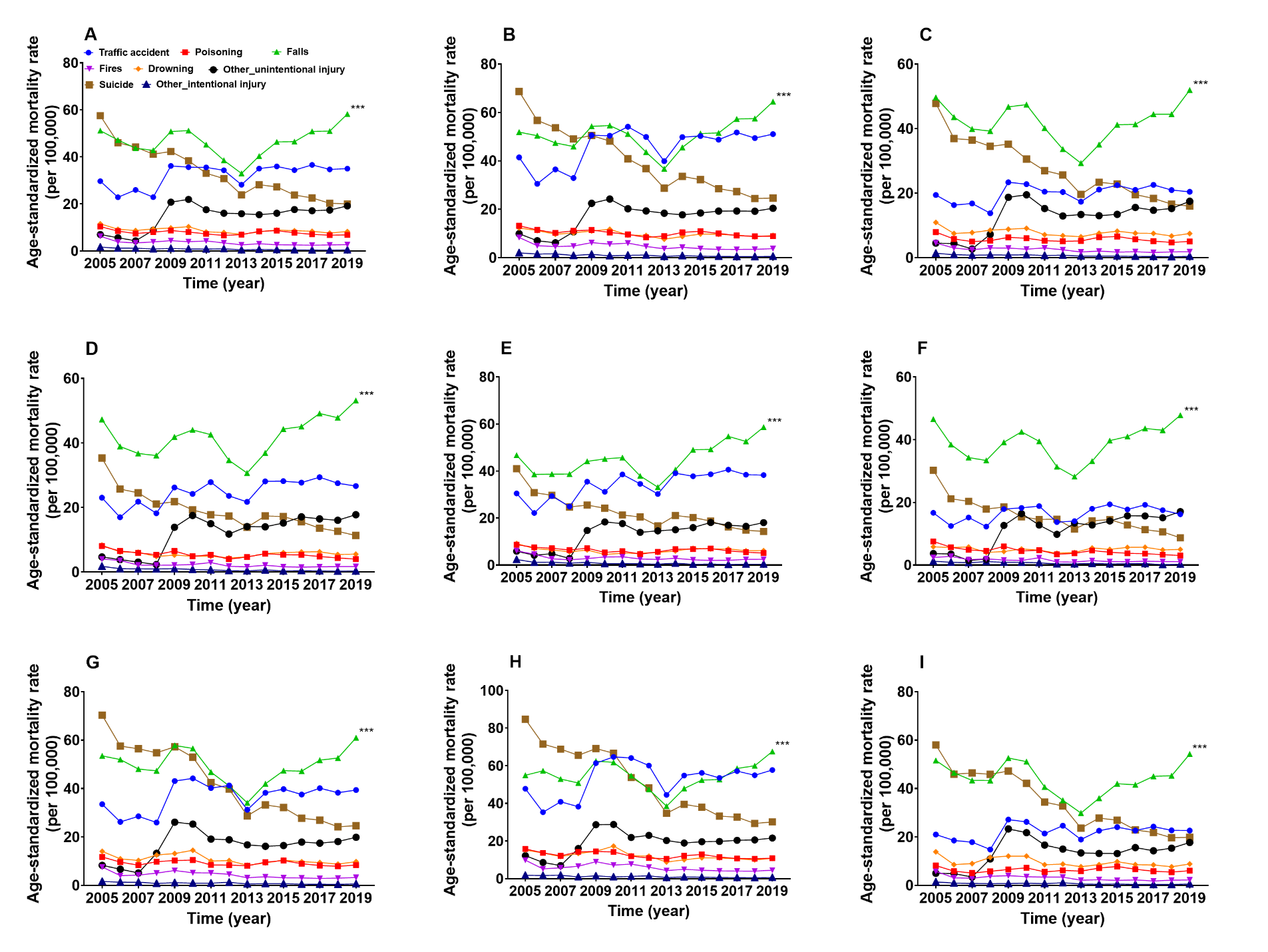


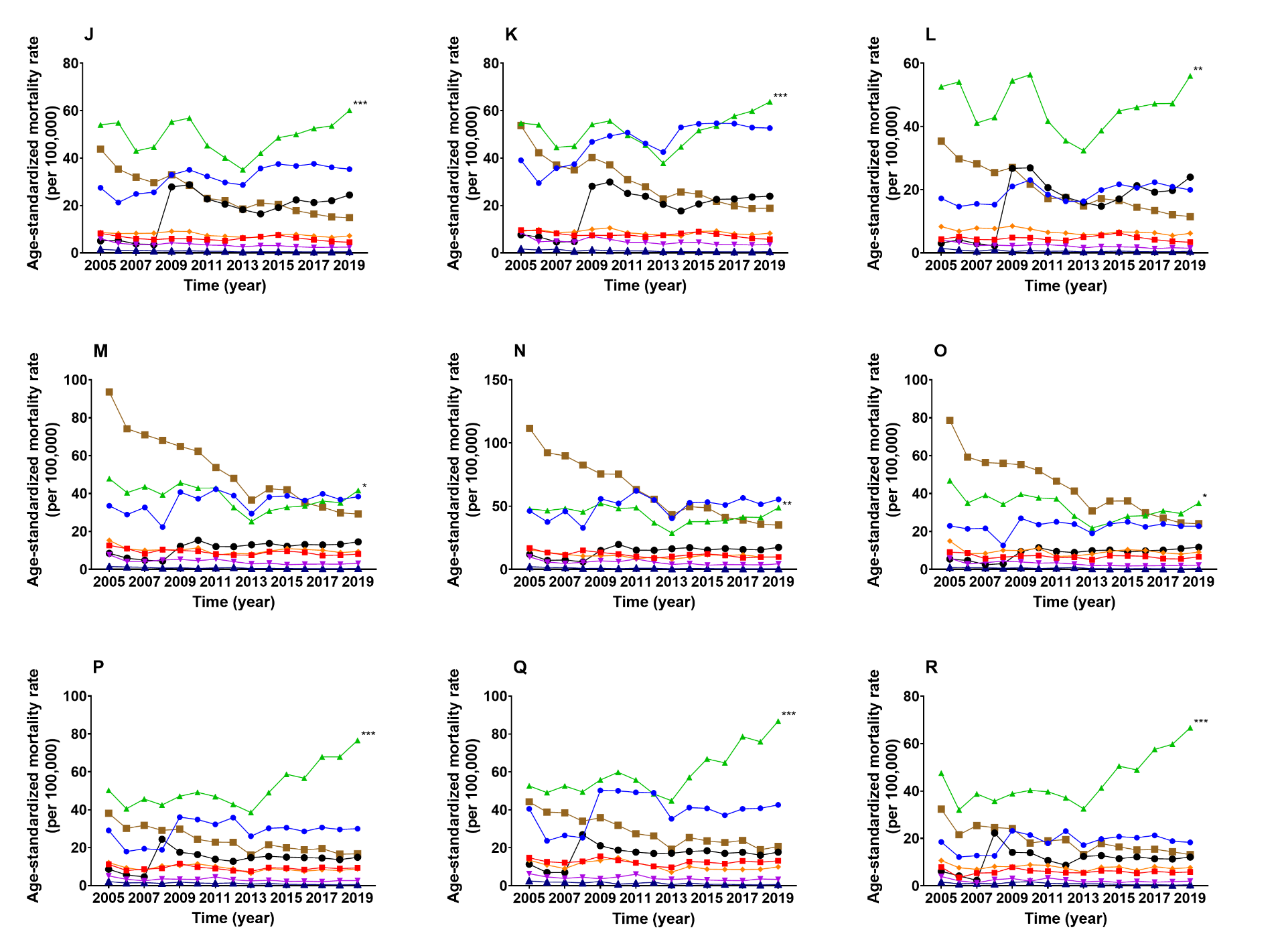


1. The trend of disparate types of injuries among people aged 65+ years in the whole population. B. The trend of disparate types of injuries among people aged 65+ years in men. C. The trend of disparate types of injuries among people aged 65+ years in women. D. The trend of disparate types of injuries among people aged 65+ years in urban. E. The trend of disparate types of injuries among people aged 65+ years in urban men. F. The trend of disparate types of injuries among people aged 65+ years in urban women. G. The trend of disparate types of injuries among people aged 65+ years in rural. H. The trend of disparate types of injuries among people aged 65+ years in rural men. I. The trend of disparate types of injuries among people aged 65+ years in rural women. J. The trend of disparate types of injuries among people aged 65+ years in eastern. K. The trend of disparate types of injuries among people aged 65+ years in eastern men. L. The trend of disparate types of injuries among people aged 65+ years in eastern women. M. The trend of disparate types of injuries among people aged 65+ years in central. N. The trend of disparate types of injuries among people aged 65+ years in central men. O. The trend of disparate types of injuries among people aged 65+ years in central women. P. The trend of disparate types of injuries among people aged 65+ years in western. Q. The trend of disparate types of injuries among people aged 65+ years in western men. R. The trend of disparate types of injuries among people aged 65+ years in western women. * *P*<.05, ** *P*<.01, *** *P*<.001 ( for trends during 2013-2019)

**Figure S4.** The trend of suicide age-standardized mortality rates among people aged 10-24 years by sex, region, and area in China, 2005-2019


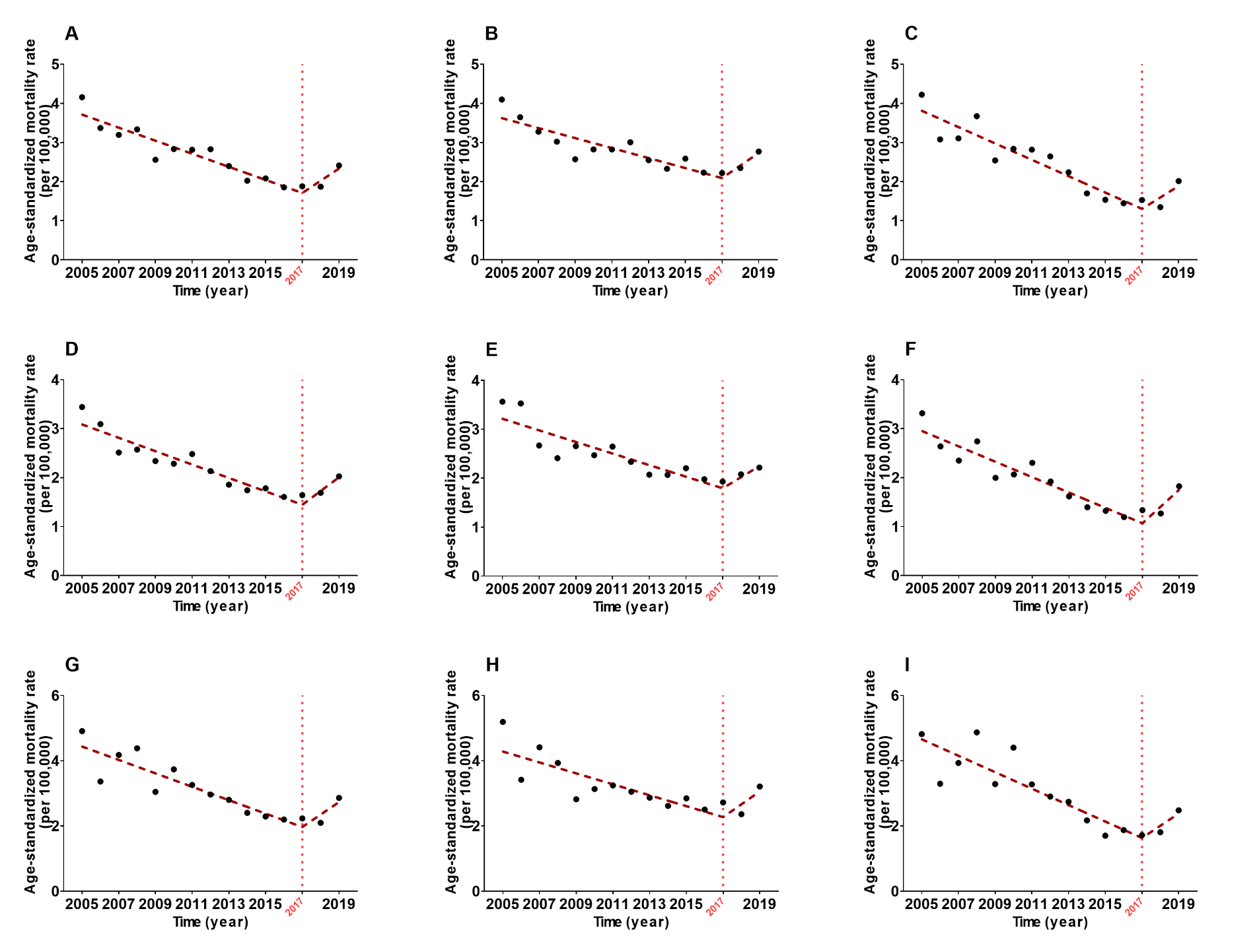


1. The trend of suicide among people aged 10-24 years in rural regions. B. The trend of suicide among men aged 10-24 years in rural regions. C. The trend of suicide among women aged 10-24 years in rural regions. D. The trend of suicide among people aged 10-24 years in central areas. E. The trend of suicide among men aged 10-24 years in central areas. F. The trend of suicide among women aged 10-24 years in central areas. G. The trend of suicide among people aged 10-24 years in western areas. H. The trend of suicide among men aged 10-24 years in western areas. I. The trend of suicide among women aged 10-24 years in western areas.

**Figure S5.** The longitudinal age trend of suicide by sex, region, and area among the 5-9 to 85+ years group in China, 2005-2019


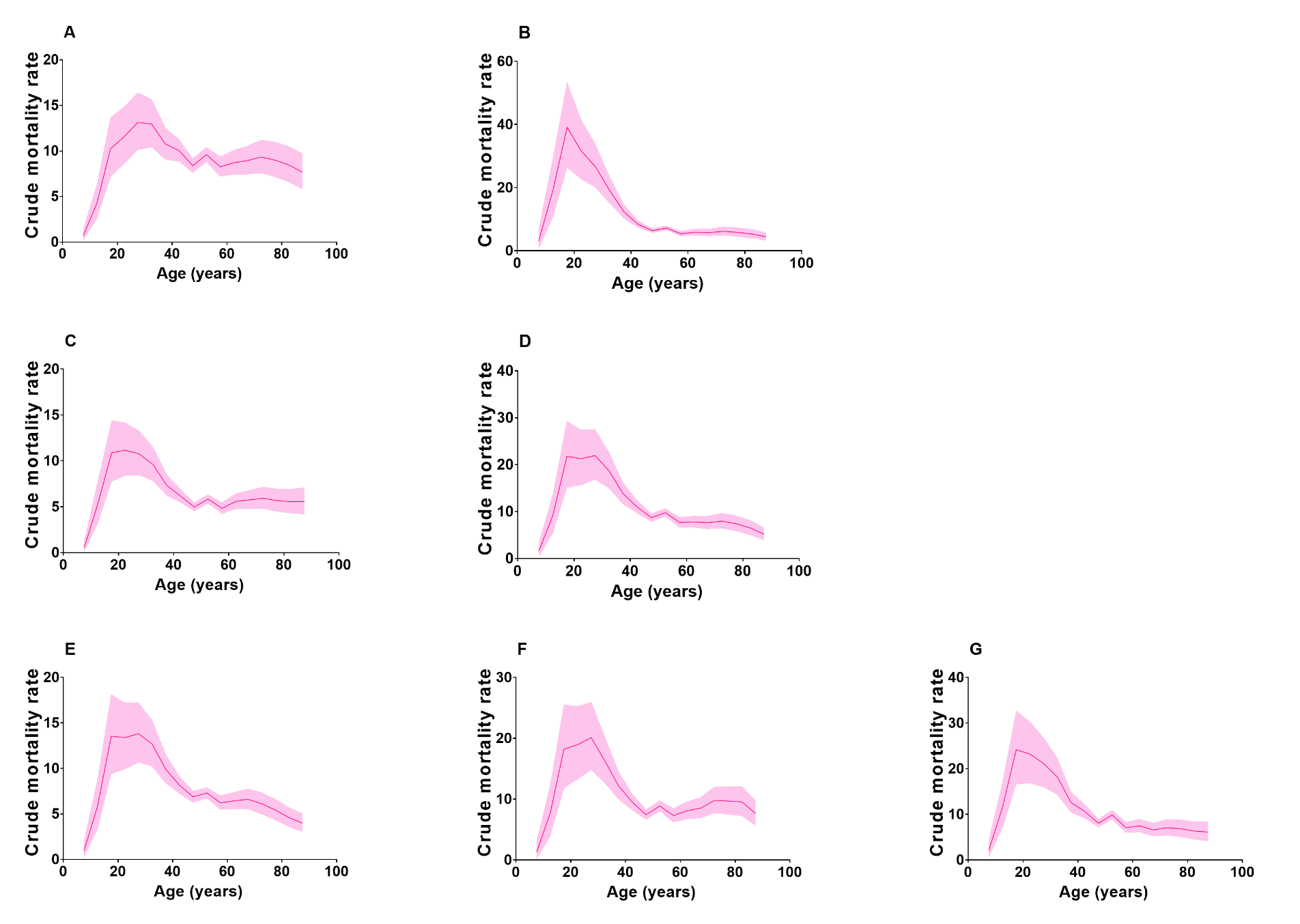


1. The longitudinal age curve of suicide in men among the 5-9 to 85+ years group. B. The longitudinal age curve of suicide in women among the 5-9 to 85+ years group. C. The longitudinal age curve of suicide in urban residents among the 5-9 to 85+ years group. D. The longitudinal age curve of suicide in rural residents among the 5-9 to 85+ years group. E. The longitudinal age curve of suicide in the east among the 5-9 to 85+ years group. F. The longitudinal age curve of suicide in the central among the 5-9 to 85+ years group. G. The longitudinal age curve of suicide in the west among the 5-9 to 85+ years group.

**Figure S6.** The trends and rank of injuries by age (≤39 years, 40-64 years, ≥65 years) among the whole study population in China, 2005-2019


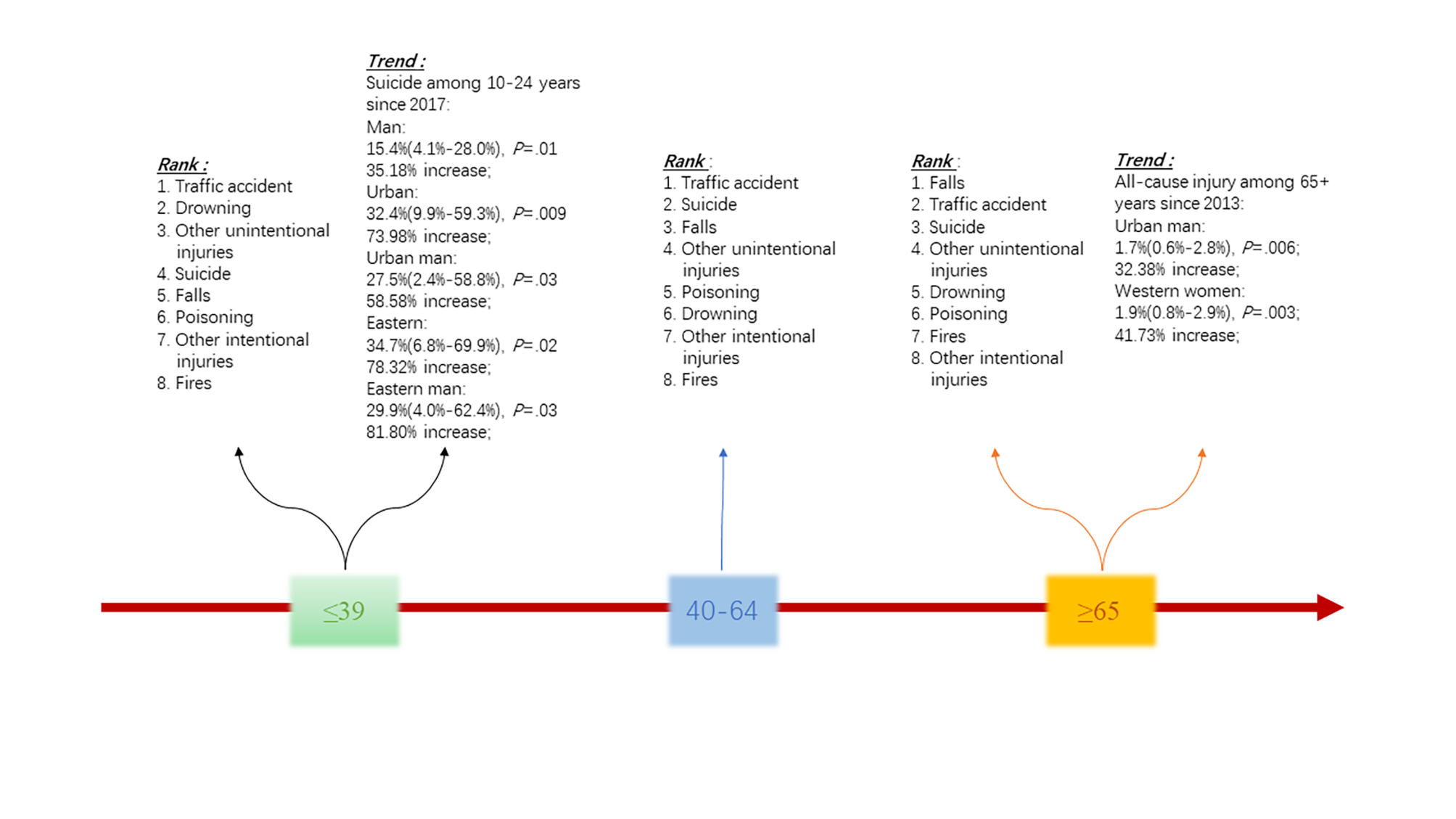

Supplement: Multimedia Appendix 2 [file publichealth_v9i1e47902_app2.docx]
